# Overexpression of improved *EPSPS* gene results in field level glyphosate tolerance and higher grain yield in rice

**DOI:** 10.1111/pbi.13428

**Published:** 2020-07-24

**Authors:** V. Mohan Murali Achary, Vijay Sheri, Mrinalini Manna, Varakumar Panditi, Bhabesh Borphukan, Babu Ram, Aakrati Agarwal, Dhirendra Fartyal, Deepa Teotia, Shyam Kumar Masakapalli, Pawan K. Agrawal, Malireddy K. Reddy

**Affiliations:** ^1^ Crop Improvement Group International Centre for Genetic Engineering and Biotechnology New Delhi India; ^2^ Odisha University of Agriculture and Technology Bhubaneswar India; ^3^ Indian Institute of Technology Mandi Kamand Mandi India

**Keywords:** EPSP synthase, glyphosate resistance, *O. sativa*, shikimate pathway, weed control, grain yield

## Abstract

Glyphosate is a popular, systemic, broad‐spectrum herbicide used in modern agriculture. Being a structural analog of phosphoenolpyruvate (PEP), it inhibits 5‐enolpyruvylshikimate 3‐phosphate synthase (EPSPS) which is responsible for the biosynthesis of aromatic amino acids and various aromatic secondary metabolites. Taking a lead from glyphosate‐resistant weeds, two mutant variants of the rice *EPSPS* gene were developed by amino acid substitution (T173I + P177S; *TIPS‐OsEPSPS* and G172A + T173I + P177S; *GATIPS‐OsEPSPS*). These mutated *EPSPS* genes were overexpressed in rice under the control of either native EPSPS or constitutive promoters (maize ubiquitin [*ZmUbi*] promoter). The overexpression of *TIPS‐OsEPSPS* under the control of the *ZmUbi* promoter resulted in higher tolerance to glyphosate (up to threefold of the recommended dose) without affecting the fitness and related agronomic traits of plants in both controlled and field conditions. Furthermore, such rice lines produced 17%–19% more grains compared to the wild type (WT) in the absence of glyphosate application and the phenylalanine and tryptophan contents in the transgenic seeds were found to be significantly higher in comparison with WT seeds. Our results also revealed that the native promoter guided expression of modified *EPSPS* genes did not significantly improve the glyphosate tolerance. The present study describing the introduction of a crop‐specific TIPS mutation in class I *aroA* gene of rice and its overexpression have potential to substantially improve the yield and field level glyphosate tolerance in rice. This is the first report to observe that the EPSPS has role to play in improving grain yield of rice.

## Introduction

Weeds, the serious biotic factor competing with crop and negatively impacting production and productivity, have been a threat to many agriculture systems. The herbicide‐tolerant (HT) technology is an integrated part of modern weed management system that has tremendously increased productivity in agriculture. Glyphosate is the most effective broad‐spectrum, non‐selective, post‐emergence systemic herbicide and facilitates economical weed control with low human and environmental risk due to its lack of residual soil activity (Funke *et al*., 2009). The herbicide, glyphosate, inhibits the shikimate pathway enzyme 5‐enolpyruvylshikimate‐3‐phosphate synthase (EPSPS, EC 2.5.1.19), by mimicking the carbocation state of PEP and reversibly binding to the enzyme in a competitive manner to form the stable but non‐covalent ternary complex EPSPS‐S3P‐glyphosate, which affects the growth of the organism by blocking the synthesis of aromatic amino acids (Priestman *et al*., 2005; Schonbrunn *et al*., 2001). The shikimate pathway is a highly conserved primitive biochemical pathway among plants, fungi and microbes and is responsible for the synthesis of chorismate, an essential precursor of both aromatic amino acids (L‐tryptophan, L‐tyrosine and L‐phenylalanine) and, many secondary metabolites (Funke *et al*., 2009). Herbicide‐resistant (HR) crops constitute more than 94% of the 180 million ha of transgenic crops grown annually worldwide and glyphosate‐resistant soybean, maize, cotton, canola and sugar beet crops have been rapidly adopted constituting 80% of those areas (Green, 2014).

Depending on their intrinsic glyphosate sensitivity and catalytic efficiency, EPSPS enzymes are categorized into two major classes (Funke *et al*., 2006). *Class I EPSPS* enzymes (glyphosate *sensitive*), which naturally occur in all plants and some bacterial species such as *E. coli*, *Klebsiella pneumoniae* and *Salmonella typhimurium* are relatively sensitive to glyphosate inhibition. On the other hand, class II EPSPS enzymes, which are found only in bacteria, *Staphylococcus aureus*, *Agrobacterium* sp. strain CP4, and *Pseudomonas* sp. strain PG2982 show both intrinsic tolerance to glyphosate and high affinity for PEP (Dill *et al*., 2008; Priestman *et al*., 2005; Tian *et al*., 2010; Tian *et al*., 2012; Tian *et al*., 2013; Wang *et al*., 2014; Zhang *et al*., 2014).

Class II EPSPS enzymes have been used mostly to develop glyphosate‐tolerant agriculturally and economically important plants (Chhapekar *et al*., 2015; Kahrizi *et al*., 2007; Yan *et al*., 2011; Ye *et al*., 2001; Zhao *et al*., 2011). In contrast, class I EPSPS enzymes are inhibited by micromolar glyphosate concentrations and have low affinity for PEP substrates (Cao *et al*., 2004; He *et al*., 2001; Pollegioni *et al*., 2011). To date, both naturally occurring class II types and mutated variants of class I types of EPSPS have been used to develop commercial glyphosate resistance (GR) crops (Funke *et al*., 2006; Funke *et al*., 2009). Significant attention has recently been given to the naturally occurring glyphosate‐sensitive class I EPSPS enzymes, as newer mutations providing increased glyphosate tolerance have been identified (Alarcón‐Reverte *et al*., 2013; Baerson *et al*., 2002; Cao *et al*., 2012; Comai *et al*., 1983; Gherekhloo *et al*., 2017; Jasieniuk *et al*., 2008; Kishore *et al*., 1986; Nandula *et al*., 2013; Padgette *et al*., 1991; Peng *et al*., 2012; Simarmata and Penner, 2008; Stalker *et al*., 1985; Sun *et al*., 2005; Tian *et al*., 2015). More recently, a T102I and P106S (TIPS) double amino acid substitution in *Eleusine indica* was identified, and the double EPSPS mutant variant was reported to provide a high level of glyphosate tolerance that was 2647‐fold greater than that of the wild‐type (WT) EPSPS and 600‐fold greater than that of the single amino acid substitution mutant (P106S) variant (Yu *et al*., 2015). Previously, the first‐generation of glyphosate‐resistant maize, trademarked as Roundup Ready (GA21 trait), utilized the rice actin 1 promoter‐driven expression of a glyphosate resistant form of maize EPSPS (i.e. TIPS‐EPSPS) (Feng *et al*., 2010) and was illegally commercialized until a new event NK603, with two copies of a slightly modified EPSPS CP4 gene was developed to improve maize tolerance to glyphosate at both vegetative and reproductive stages (Green and Castle, 2010). However, in the majority of cases, transgenic overexpression of class I EPSPS mutant variant fails to result in satisfactory glyphosate resistance for commercial applications. This is because most of the class I EPSPS mutants display glyphosate resistance at higher PEP Km values, which affects the interaction between EPSPS and PEP, thereby slowing down the rate of reaction at low PEP concentrations in the chloroplasts (Bradshaw *et al*., 1997). Therefore, obtaining novel and functional glyphosate‐tolerant *EPSPS* genes that have multiple site mutations targeting other amino acid residues in the enzyme is essential for future crop improvement programmes.

Previously, we have characterized the P177S substitution mutation in transgenic rice which showed moderate level of resistance to glyphosate (Chandrasekhar *et al*., 2014). Similarly, we have also used the combination strategy and developed transgenic rice plant expressing both glyphosate‐tolerant rice *EPSPS* gene (P–S) and glyphosate‐detoxification *igrA* gene (Vemanna *et al*., 2017), where the transgenic plants were endowed with improved tolerance to glyphosate (Fartyal *et al*., 2018). In the present study, we introduced multi‐amino acid substitutions in *OsEPSPS* to develop field level glyphosate‐tolerant rice plants. The constitutively overexpressed *TIPS‐OsEPSPS* with double substitution mutation enabled the rice plants to develop significantly greater resistance to higher doses of glyphosate applications both under controlled and field conditions. Thus, the approach reported here is quite promising for the introduction of TIPS mutations into other elite crops either by gene transformation or by conventional breeding in conjunction with marker‐assisted selection approaches for developing glyphosate‐tolerant crop varieties for modern agriculture.

## Results

### *In silico* analysis and identification of glyphosate‐tolerant mutations

**Figure 1:**
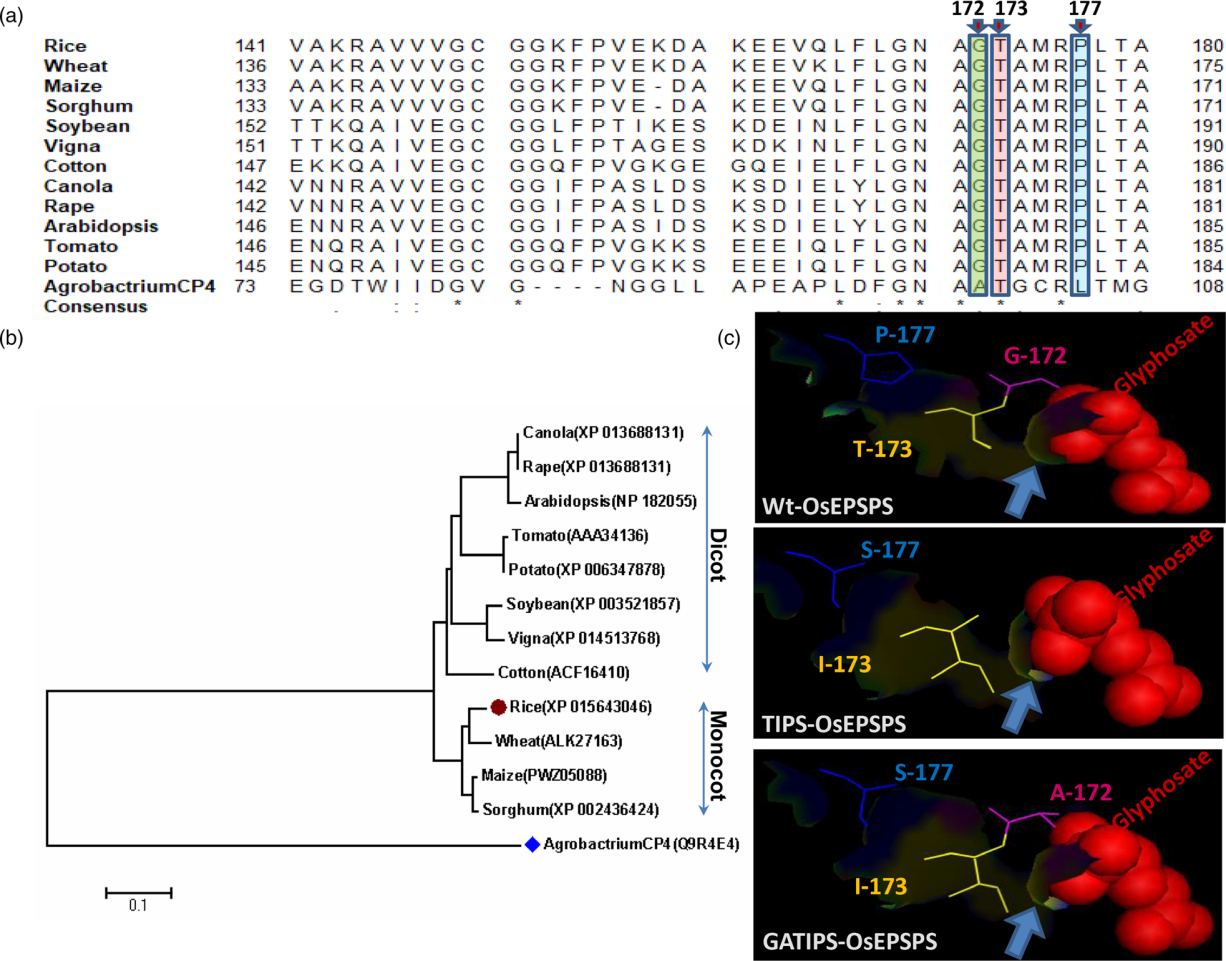
*In silico* determination of conserved amino acids in EPSPS proteins belonging to various organisms, determination of phylogenetic relationship between them and structure prediction of rice EPSPS protein. Sequence alignment of EPSPS among the plants and *Agrobactrium* to identify the conserved amino acid proline (P), tyrosine (T) and Isoleucine (I). (a) The arrow indicates the conserved G‐172, T‐173 and P‐177 in rice EPSPS protein. (b) Phylogenetic analysis of EPSPS proteins among the plants. (c) Predicted protein structure showing mutation points and interaction of glyphosate in the rice EPSPS active site.

The overuse of glyphosate has resulted in the evolution of GR weed biotypes. This herbicide selection pressure leads to the accumulation of favourable mutations in the active site of the target enzyme (Figure 1c), which makes the enzyme resistant to glyphosate. Homology searches, multiple sequence alignments and phylogenetic analysis of protein sequences from different organisms revealed that the rice EPSPS enzyme shared 76%–89% sequence similarity with other class I plant EPSPS and 21% with *Agrobacterium*‐CP4 class II EPSPS protein (Figure 1a,b; Table S3). Further, the sequence analysis of plant EPSPS also revealed that the amino acids G‐172 T‐173 and P‐177 (in rice EPSPS) were well conserved among the monocots and dicots (Figures 1a).

### Development and molecular confirmation of rice events

**Figure 2:**
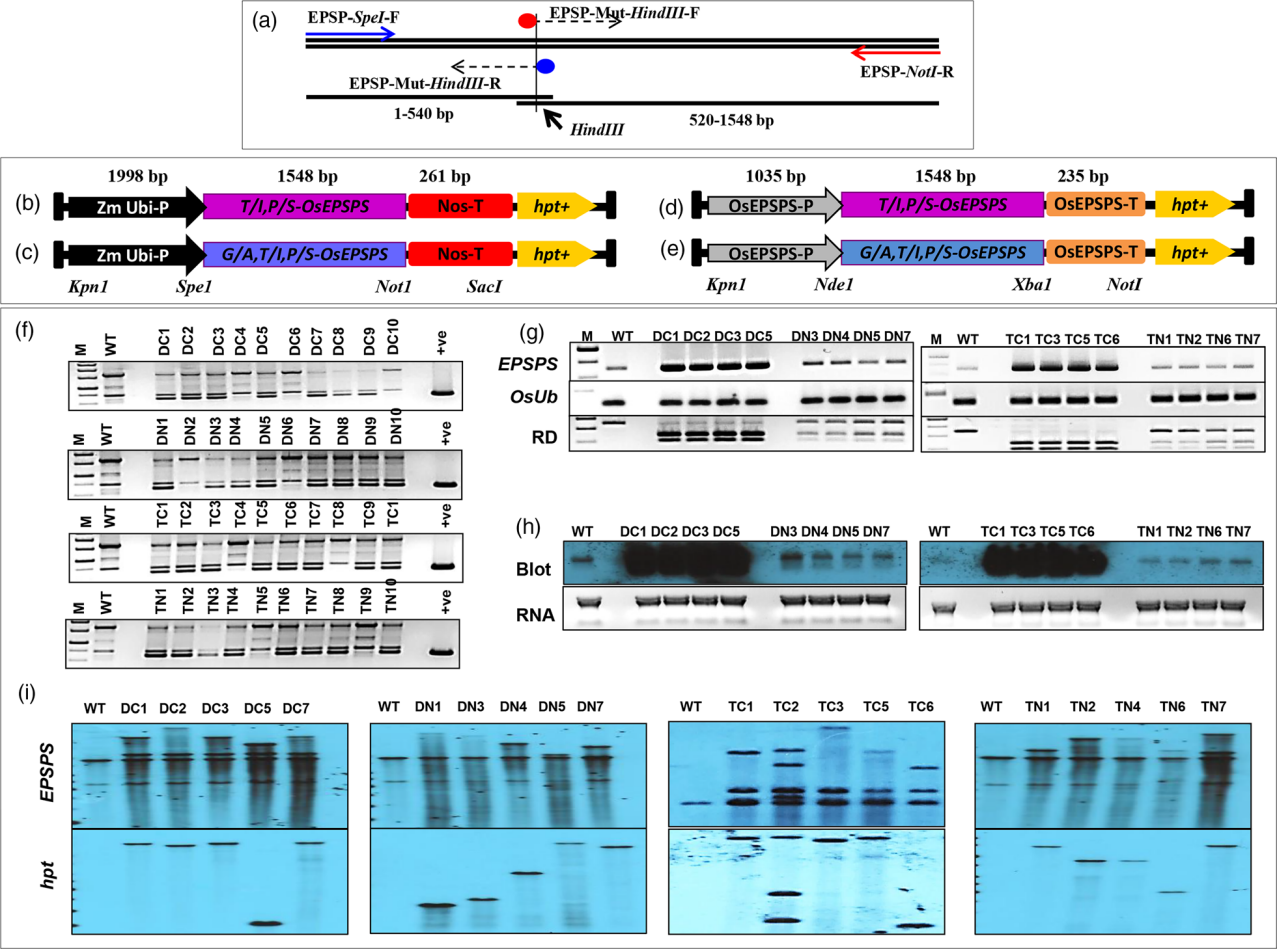
Construction of plant expression cassette and molecular analysis of overexpressed rice plants. (a) PCR‐mediated introduction of point mutations in the rice *EPSPS* gene. Double (*TIPS‐OsEPSPS*) or triple (*GATIPS‐OsEPSPS*) mutations in the rice *EPSPS* gene (*OsEPSP*S) were introduced *via* PCR, in which the EPSP‐Mut‐*Hind*III‐F and EPSP‐Mut‐*Hind*III‐R primers contained altered nucleotide sequences along with a restriction site for *Hind*III. The rice double‐mutant (*TIPS‐OsEPSPS*) and triple‐mutant (*GATIPS‐OsEPSPS*) EPSPS expressed either under the control of the ZmUbi promoter (b and c) or under the regulation of the native rice EPSPS promoter (d and e). These expression cassettes in a pMDC99 vector were used to transform rice calli. (f) Screening of transformed rice plants with EPSPS primers for the identification of positive rice plants, where ‘M’ denotes ‘1 kb ladder’, ‘WT’ denotes ‘wild type or control plant’, ‘DC’ denotes ‘double mutant under constitutive promoter’, ‘DN’ denotes ‘double mutant under native promoter’, ‘TC’ denotes ‘triple mutant under constitutive promoter’, ‘TN’ denotes ‘triple mutant under native promoter’ and ‘+ve’ denotes ‘positive control’. (g) Semiquantitative RT‐PCR to determine and compare the expression of the *EPSPS* genes between WT and various overexpressed rice lines. Semiquantitative RT‐PCR was also carried out with rice *ubiquitin* primers (*OsUb*) as an internal control. The PCR‐amplified EPSPS transcripts were also restriction digested (RD) with the *Hind*III enzyme to visualize and compare the expression of native and overexpressed *EPSPS* copies in the rice lines. (h) Northern blots with *EPSPS* probes in WT and overexpressed rice lines. (i) Southern blots with *EPSPS* and hygromycin (*hpt*) to determine the copy number of overexpressed rice lines.

Regulation of gene expression during various developmental stages and under various environmental conditions is of paramount importance for improving the agronomic traits of plants. Hence, the choice of promoter is an important component of gene expression. A strong promoter driving transgene expression results in better physiological performance of the transgenic plants under various circumstances. Since EPSPS is a part of the shikimate pathway and synthesizes many metabolites including essential aromatic amino acids, we chose to overexpress the mutated *TIPS‐OsEPSPS* (Figure 2a; Figure S4) and *GATIPS‐OsEPSPS* (Figure 2a; Figure S5) genes under the control of two different promoter systems, the maize ubiquitin promoter (*ZmUbi* promoter; Figure S2) and native promoter of *OsEPSPS* (Figure 2b‐e; Figure S1) to impart a high level of glyphosate tolerance and to improve the physiology of the overexpressed plants. Since glyphosate inhibits the EPSPS enzyme in the plant chloroplast‐localized shikimate pathway, presence of chloroplast transit peptide in the gene is a necessity for developing transgenic plants. Hence, in this study, we have used the full‐length coding sequences of mutated variants of EPSPS, containing chloroplast transit peptide (amino acid 1–70; Figure S3) for targeting the proteins to chloroplast.

The individual expression cassettes of *TIPS‐OsEPSPS* and *GATIPS‐OsEPSPS* were generated separately in pEV‐1 (entry vector 1 of Gateway system, LifeTech) and subsequently transferred into the plant transformation vector pMDC99 by the LR‐gateway cloning method. Overexpressed transgenic rice lines (Japonica, variety Nipponbare) were developed *via A. tumefaciens‐*mediated plant transformation. T1 lines were selected by using hygromycin in the selection media. The integration of the transgenes was confirmed by PCR using *EPSPS* gene‐specific primers (Figure 2f). Approximately 90% of the putative transformants were found to contain the transgenes. The mutant variants of the EPSPS transgene were further distinguished from native EPSPS copies by restriction digestion of PCR‐amplified *EPSPS* gene products *via* the *Hind*III enzyme (details described in materials and methods section).

The copy number of the transgene was determined by Southern blotting with *hpt* and *EPSPS* gene‐specific probes (Figure 2i). The T1 lines presented double (for single transgene copy integration) or multiple (for more than one transgene copy integration) hybridization band signals when *EPSPS* gene‐specific probes were used for hybridization, and WT non‐transformed plants presented single *EPSPS* hybridization signals. However, hybridization with the *hpt* gene probe resulted in the display of one (for single transgene copy integration) or more (for more than one transgene copy integration) distinct signals in the T1 rice lines, while the genomic DNA of the WT plants did not exhibit any signal (Figure 2i). Single copy integrated rice lines from the *TIPS‐OsEPSPS* and *GATIPS‐OsEPSPS* under the native *EPSPS* promoter and constitutive *ZmUbi* promoter were selected for further analysis. The expression levels of the transgene(s) were confirmed by northern blot analysis and semiquantitative reverse transcription‐PCR (RT‐PCR) in the T2 homozygous transgenic lines (Figure 2g,h). The expression analysis revealed that, compared with the native *EPSPS* promoter, the constitutive *ZmUbi* promoter resulted in a higher level of *TIPS‐OsEPSPS* and *GATIPS‐OsEPSPS* expression (Figure 2g,h). The PCR amplicons obtained from the RT‐PCR analysis were further digested with the *Hind*III restriction endonuclease to distinguish native EPSPS transcripts from the transgenic ones (Figure 2g). Northern blotting with *EPSPS* gene‐specific probes revealed a significantly higher level of *TIPS‐OsEPSPS* and *GATIPS‐OsEPSPS* expression when expressed under the regulation of the ZmUbi constitutive promoter (Figure 2h). Primers used for various purposes of the present study are depicted in Table S1.

### Physiological characterization of the overexpressed rice lines

**Figure 3:**
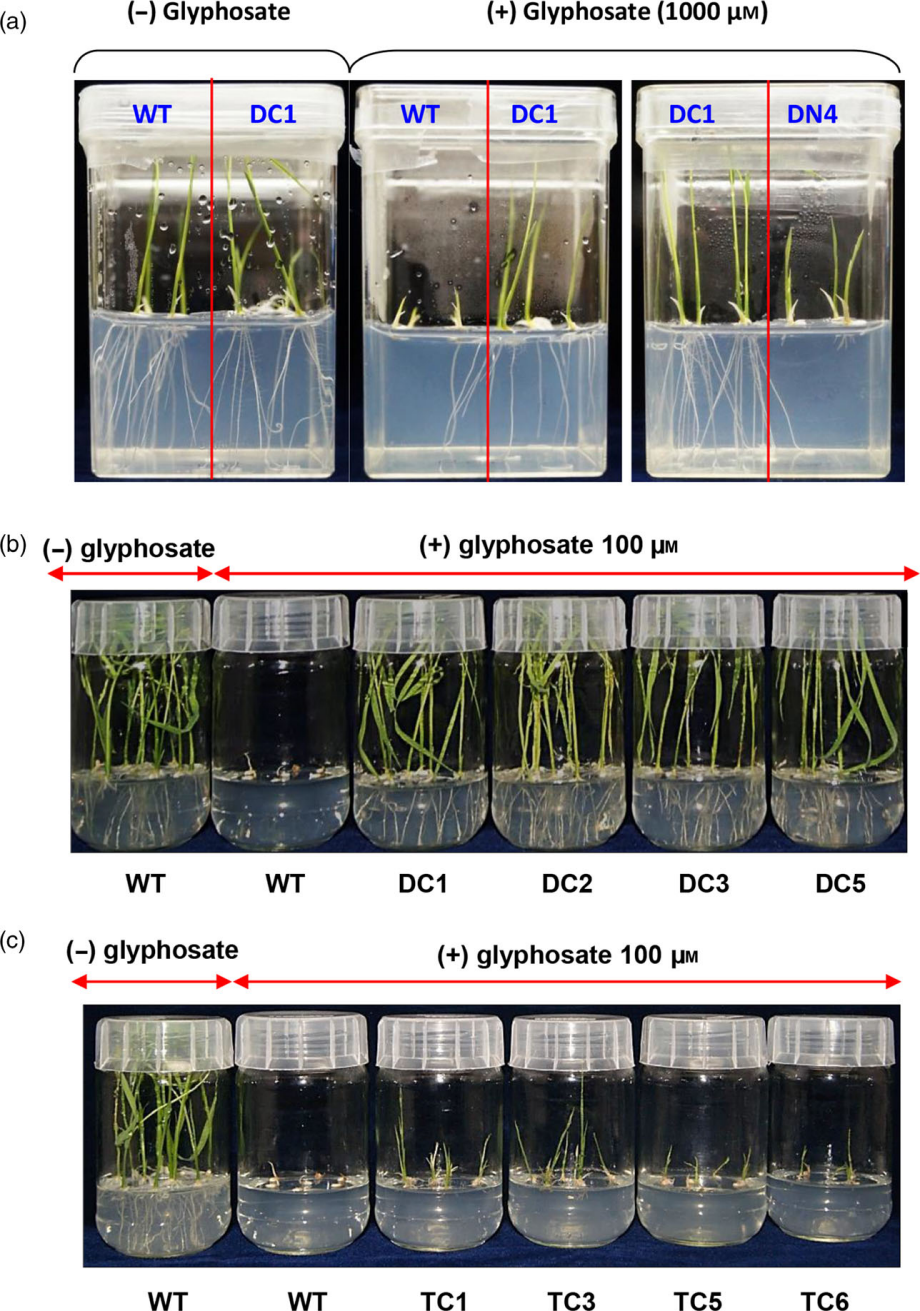
Seedling germination and growth assays in response to glyphosate treatment. (a) Comparison of the growth of WT, DC and DN seedlings in media supplemented with 1 mm glyphosate. (b) Screening the performance of various lines of DC plants via their germination on media supplemented with 100 µm glyphosate. (c) Screening the performance of various lines of TC plants via their germination on media supplemented with 100 µm glyphosate.

We used homozygous and stabilized T2 transgenic lines for carrying out all the physiological assays. For the seedling growth assays, seeds from WT and various T2 transgenic rice lines expressing *TIPS‐OsEPSPS* double mutant (DC; double‐mutant constitutive promoter and DN; double‐mutant native promoter) and *GATIPS‐OsEPSPS* triple mutant (TC; triple‐mutant constitutive promoter, and TN; triple‐mutant native promoter) were surface sterilized and directly placed on half‐strength MS media supplemented without or with 100 µm glyphosate within the glass bottles. The germinated seeds were allowed to grow for 15 days in the growth chamber under controlled environmental conditions (25°C, 12:12 h light/dark photoperiod), and the plant growth parameters were imaged and recorded. The DN rice lines had significantly less growth than their DC counterparts when grown in the presence of glyphosate (Figure 3a). As evident in Figure 3b, in the presence of 100 µm glyphosate, the WT rice seeds either failed to germinate or experienced severe growth inhibition and appeared brown in colour. In contrast, each of the four DC rice lines (DC1, DC2, DC3 and DC5) showed robust growth even in the presence of glyphosate at concentrations, toxic to WT rice. The glyphosate‐treated T2 rice lines exhibited growth comparable to that of untreated WT seedlings in terms of root and shoot development and leaf greenness. These data showed that the expression of mutant rice EPSPS lines harbouring twin mutations (T173I and P177S) resulted in tolerance to glyphosate toxicity at levels that are detrimental to WT rice plants.

TC‐overexpressed rice lines were also confirmed for their level of glyphosate tolerance. As evident from Figure 3c, compared with WT plants grown in the absence of glyphosate treatment, WT rice in the presence of 100 µm glyphosate died after germination. In contrast, each of the four TC rice lines (TC1, TC3, TC5 and TC6) exhibited marginal root development and stunted shoot development with moderately green shoots. The tolerance to glyphosate exhibited by the TC plants was marginal (Figure 3c). Compared with that of the WT rice EPSPS, the glyphosate tolerance capability of the triple‐mutant variant of rice EPSPS exhibited minimal improvement and was substantially observed to be lower than that of the double‐mutant variant or that of the DC plants (Figure 3b,c).

These data, shown in Figure 3b,c, collectively suggest that the overexpression of double‐mutant variants (P177S and T173I) of the EPSPS protein in rice provides increased tolerance to glyphosate at a high level of dosage. However, the introduction of an additional mutation (G172A) disrupted the glyphosate tolerance level, and such overexpressed lines did not exhibit any significant improvement in the level of glyphosate tolerance.

**Figure 4:**
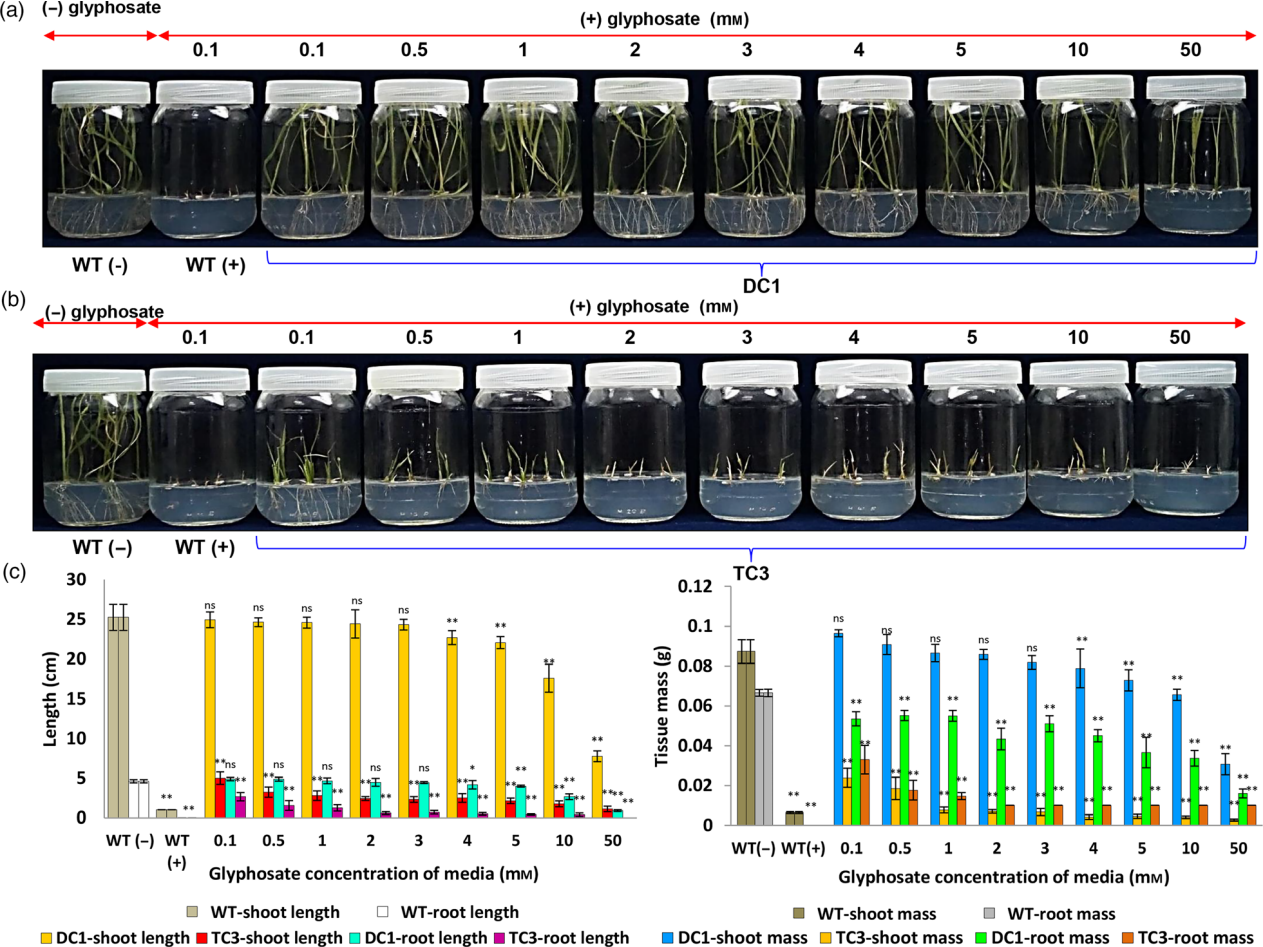
Germination and growth analysis of DC and TC lines under increasing concentrations of glyphosate. (a) DC1 plants could germinate and grow well in a wide range of glyphosate‐supplemented media. (b) Growth of TC1 plants was highly compromised with an increase in glyphosate concentration in the media. (c) Graphs depicting the shoot and root fresh weights and lengths of the overexpressed plants grown in media supplemented with increasing concentrations of glyphosate. The increasing glyphosate concentrations significantly [*P* ≤ 0.05 (*) or *P* ≤ 0.01 (**)] inhibited the shoot‐root length and biomass of DC1 and TC3 lines at level compared to their respective WT (−) controls. The experiment having 10 biological replications was repeated in triplicates. The ns symbol represents non‐significant.

Seeds from DC1 and TC3 lines were germinated and grown continuously in the presence of increasing concentrations of glyphosate (0.1–50 mm), and it was found that the DC1 rice lines could grow in media that contained very high levels of glyphosate (3 mm) without any significant loss of shoot and root length or shoot mass. In contrast, TC3 rice lines had significantly (*P* ≤ 0.01) reduced shoot and root length, shoot and root mass even at lower concentrations (i.e. 0.1 mm onward) glyphosate treatment (Figure 4a‐c). These findings indicated that constitutive overexpression of *TIPS‐OsEPSPS* gene results in very high level of tolerance to glyphosate.

**Figure 5:**
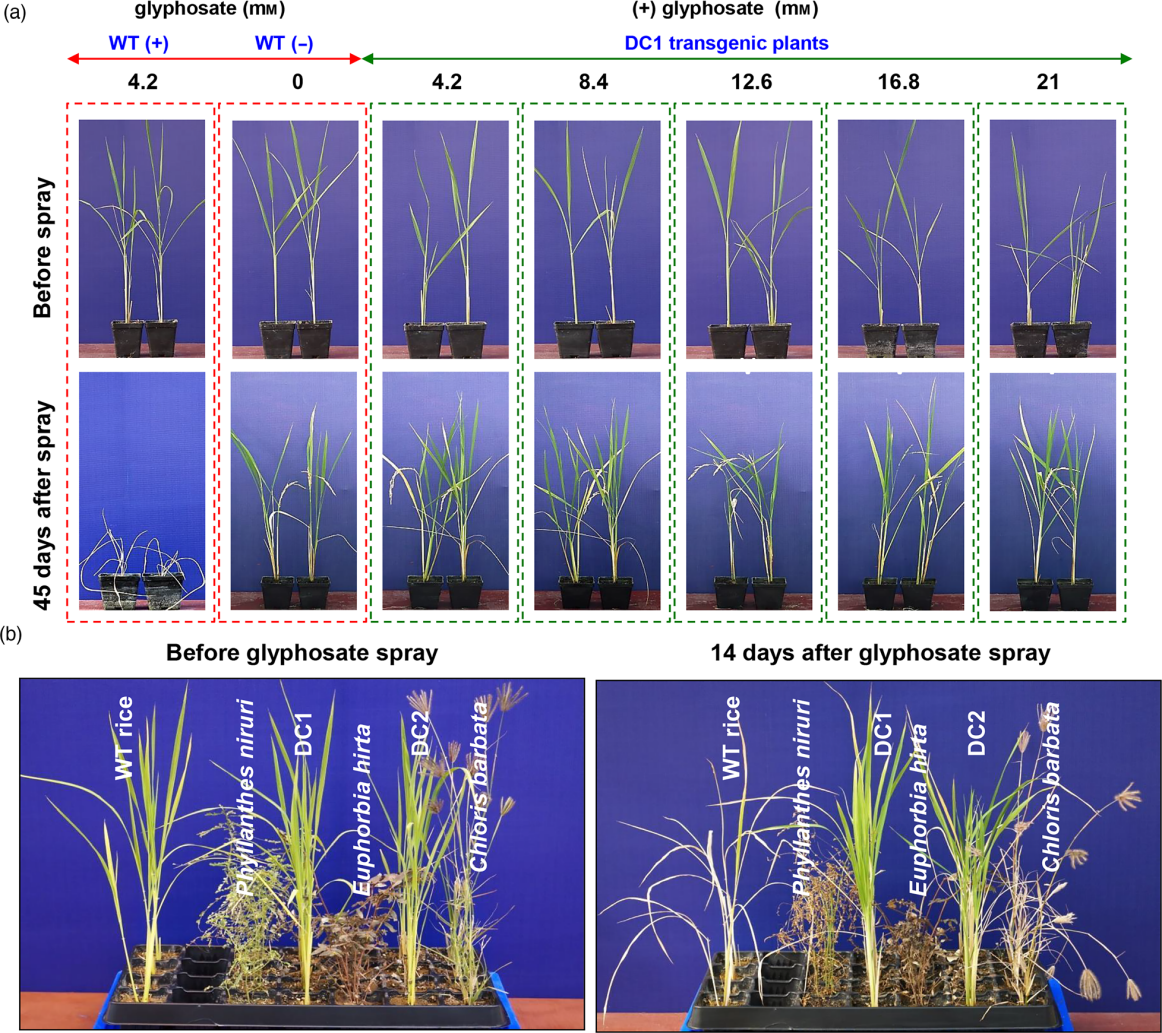
Evaluation of glyphosate on WT plant and DC lines and weed competition assays. (a) Various doses of glyphosate were tested by spraying the herbicide on WT and DC1 plants. The DC1 lines could tolerate and produce panicles when sprayed with both relatively low and high doses of glyphosate, while the WT plants died in response to low doses of glyphosate spraying. (b) Figures represent the post‐emergent herbicidal action of glyphosate before and after foliar applications (6.3 mm) on weeds, WT plants and DC rice lines.

To test the effects of various doses of commercially available glyphosate (41% glyphosate, Roundup Ready, Monsanto) on the growth of WT and DC lines, rice seedlings were grown in soil pots. Twenty‐day‐old WT and DC1 rice lines were sprayed without or with glyphosate at concentrations of 4.2, 8.4, 12.6, 16.8 and 21 mm, and the growth and morphological characteristics of the plants were periodically recorded until the seeds matured. As evident in Figure 5a, under normal conditions (without glyphosate application), the growth of both WT and DC were comparable. Glyphosate at concentration of 4.2 mm had a detrimental effect on WT plants and the plants died within 14 days after glyphosate application. On the other hand, glyphosate concentrations of 4.2, 8.4, 12.6, 16.8 and 21 mm had no detrimental effect on the growth of the DC1 plants, showing a high level of tolerance to glyphosate. Furthermore, the glyphosate‐sprayed DC1 plants were periodically monitored until maturity, and it was observed that they exhibited normal growth and morphological characteristics similar to those of the WT plants, not sprayed with glyphosate.

For the crop–weed competition experiment, WT plants and DC1 and DC2 as well as weeds (*Phyllanthus niruri*, *Euphorbia hirta* and *Chloris barbata*) were grown in treatment tray that contained soil and vermiculite mixture (1:2 v/v ratio) in the greenhouse for 2 weeks and subsequently 6.3 mm glyphosate solution was sprayed once on the seedlings. The plants were photographed on 14th day of glyphosate application (Figure 5b). The experiment demonstrates that, the DC lines had healthy growth and were not affected by glyphosate treatment. In contrast, the WT rice and weeds appeared completely brown and subsequently died. The results suggest that the DC lines had positive response to glyphosate application and were effective in weed management to eliminate different weed biotype in rice cultivation.

**Figure 6:**
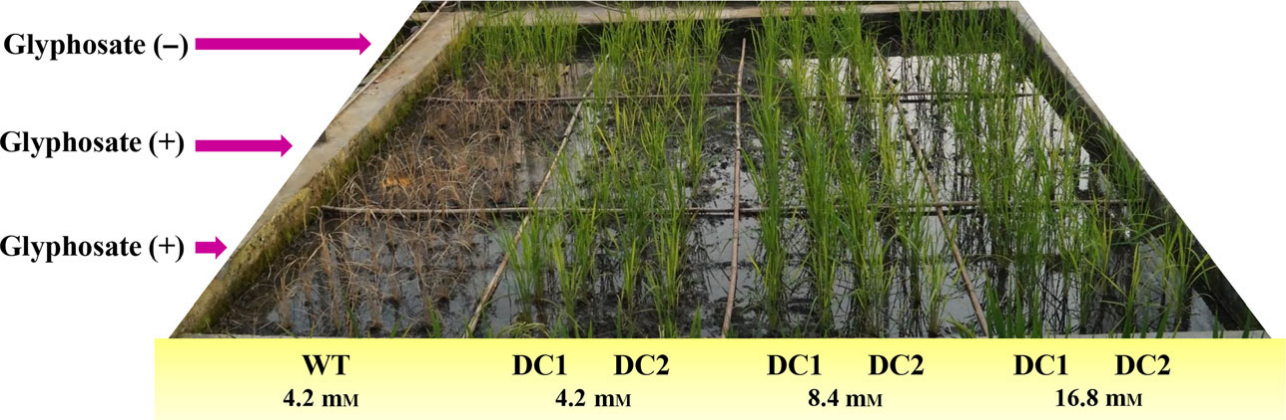
Field experiments to determine the glyphosate tolerance of DC rice lines. The DC lines could tolerate and set seeds well when sprayed with glyphosate (4.2, 8.4 and 16.8 mm) under field conditions.

The overall productive fitness and yield of the DC lines were evaluated in the control and glyphosate treatments under field conditions (Figure 6; Table 1a and b). Glyphosate spraying resulted in the complete death of the WT seedlings (at 4.2 mm spraying concentration), while the DC1 and DC2 seedlings grew normally without any loss of vigour or yield (at 4.2, 8.4 and 16.8 mm spraying concentration) (Figure 6; Table 1a). The DC lines could tolerate up to fourfold (16.8 mm) that of the dose which killed the WT plants. Among various yield parameters studied, number of tillers per plant, single seed weight and 100 grain weight of transgenic lines (DC and TC lines) were comparable to those of WT plants that were not treated with glyphosate. However, DC1 transgenic lines had significantly improved number of seeds/plant despite of glyphosate spaying at 4.2 and 8.4 mm concentrations and there was significantly higher total yield/plant in case of DC1 plants with 4.2 mm glyphosate application (Table 1a). Similarly, DC2 transgenic lines had significantly improved number of seeds/plant and total yield/plant even after 4.2 and 8.4 mm glyphosate spraying. Furthermore, the DC lines under field condition, without glyphosate treatment showed robust growth and produced significantly (*P* ≤ 0.01) higher number of seeds/plant and their total grain yield/plant increased by 17‐19% compared to WT control plant (Table 1b).

**Table 1:** (a) Yield parameters of DC lines after glyphosate spraying under field conditions. (b) Comparative yield parameters of overexpressed rice lines (DC and TC) under controlled confined field conditions

Treatments	Plant height (cm)	No of tiller/plant	Main panicle length (cm)	Number of seeds/plant	Seed weight (mg)	100 grain weight (g)	Total yield/plant (g)
WT (4.2 mm)	All plants died						
WT (0 mm)	70.23 ± 1.16	6.75 ± 0.45	15.65 ± 0.55	410.67 ± 21.85	24.89 ± 1.39	2.26 ± 0.11	10.45 ± 1.05
DC1 (4.2 mm)	71.49 ± 1.61	6.92 ± 0.29	16.91 (**) ± 1.52	465.17 (**) ± 46.49	25.43 ± 1.22	2.24 ± 0.11	11.84 (*) ± 1.42
DC1 (8.4 mm)	70.57 ± 1.20	6.83 ± 0.39	16.28 ± 0.94	456.25 (*) ± 39.61	24.98 ± 1.53	2.18 ± 0.05	11.39 ± 1.41
DC1 (16.8 mm)	69.38 ± 0.98	6.67 ± 0.49	15.63 ± 0.77	412.75 ± 34.93	24.78 ± 0.62	2.13 ± 0.15	10.23 ± 0.97
DC2 (4.2 mm)	72.06 ± 2.93	7.17 ± 0.39	17.25 (**) ± 1.82	470.75 (**) ± 30.25	25.53 ± 1.55	2.29 ± 0.13	12.02 (**) ± 1.13
DC2 (8.4 mm)	71.00 ± 1.09	7.00 ± 0.43	17.15 (*) ± 1.55	464.67 (**) ± 54.95	25.15 ± 1.07	2.22 ± 0.06	11.67 (*) ± 1.31
DC2 (16.8 mm)	70.03 ± 0.95	6.83 ± 0.39	16.88 (*) ± 0.95	434.58 ± 44.54	24.89 ± 0.83	2.17 ± 0.06	10.81 ± 1.09

	Plant height (cm)	No of tiller/plant	Main panicle length (cm)	Number of seeds/plant	Seed weight (mg)	100 grain weight (g)	Total yield/plant (g)
WT	70.17 ± 2.18	6.83 ± 0.39	15.42 ± 1.0	405.08 ± 26.08	25.42 ± 2.45	2.29 ± 0.24	10.31 ± 1.24
TC1	70.12 ± 1.99	6.83 ± 0.39	16.21 ± 1.13	419.83 ± 42.44	25.55 ± 2.81	2.32 ± 0.16	10.74 ± 1.66
TC3	70.59 ± 1.92	6.83 ± 0.39	16.36 ± 1.12	416.50 ± 52.49	25.50 ± 1.42	2.29 ± 0.11	10.62 ± 1.51
DC1	73.33 ± 1.97	7.08 ± 0.29	17.03 (**) ± 1.02	474.33 (**) ± 47.93	25.54 ± 1.40	2.29 ± 0.15	12.10 (**) ± 1.27 (17% ↑ than WT)
DC2	74.12 ± 5.22	7.00 ± 0.43	17.29 (**) ± 1.41	477.75 (**) ± 38.12	25.80 ± 1.58	2.31 ± 0.07	12.33 (**) ± 1.26 (19% ↑ than WT)

The agronomic traits including main panicle length, number of seeds per plant and grain yield per plant increased significantly at *P* ≤ 0.05 (*) or 0.01 (**) compared to the WT plants. Data are means ± SD (12 plants).

**Figure 7:**
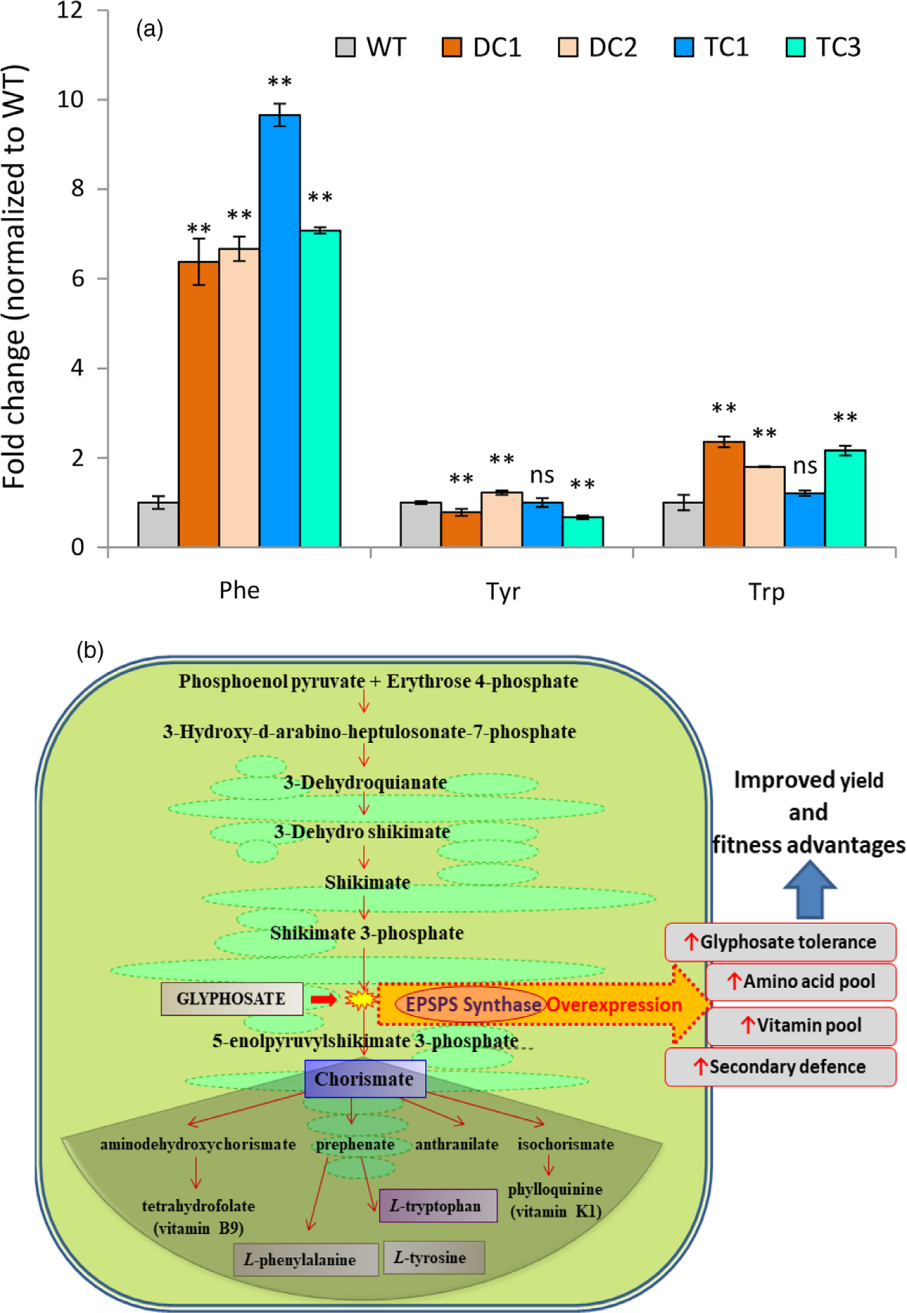
Quantification of aromatic amino acid level in WT and transgenic lines. (a) Relative fold changes of free aromatic amino acids – phenylalanine, tyrosine and tryptophan in transgenic rice seeds increased significantly at *P* ≤ 0.01 (**) in relation to WT (set at 1). The amino acids in the rice seeds were analysed by GC‐MS after TBDMS derivatization. The analysis shows that transgenic lines have higher accumulation of phenylalanine (>6 fold change). A twofold increase in tryptophan levels were observed in transgenic lines of DC1, DC2, and TC3. Tyrosine levels were variable with marginal level changes in relation to the WT. See Figure S7 for relative peak abundances normalized to internal standard norleucine. Data are means ± SD (from 3 independent plants). (b) Schematic diagram showing that modifications to the shikimate pathway can potentially improve glyphosate tolerance as well as amino acid, vitamin and secondary metabolite pools in plants.

The EPSP synthase is the key enzyme for biosynthesis of aromatic amino acid pool in the plants. We quantified the phenylalanine, tyrosine and tryptophan contents in seeds of the DC1, DC2, TC1 and TC3 plants using gas chromatography–mass spectrometry (GC‐MS). Interestingly, we noticed that the phenylalanine and tryptophan content of these seeds significantly increased in comparison with the WT seeds. The DC seeds on an average had about 6.5‐fold higher phenylalanine content while in the TC seeds, the phenylalanine content was about 8.4‐fold higher in comparison with WT seeds. Further, DC seeds had about twofold higher content of tryptophan while the TC3 seeds had about 2.2‐fold higher content of tryptophan as compared to WT seeds. The TC1 seeds had non‐significant increase in the tryptophan level. However, the tyrosine levels of the said transgenic lines did not follow any pattern. For example, while tyrosine level in the DC1 seeds decreased, it increased in case of the DC2 seeds, and while tyrosine content of the TC1 seeds remained unchanged, it significantly decreased in case of TC3 seeds. Overall, the results indicated that *ZmUbi* promoter guided overexpression of modified EPSPS enzymes resulted in significant increase in phenylalanine content in the seeds of the transgenic lines by a huge margin (Figure 7a).

It can be concluded from the present study that the *TIPS‐OsEPSPS* under the strong constitutive promoter provided high degree of glyphosate tolerance under field conditions. The DC lines overexpressing EPSP synthase in the absence of glyphosate application showed fitness advantages and resulted in increased grain yield and aromatic amino acid content (phenylalanine and tryptophan) of the transgenic rice plants.

## Discussion

The crop productivity is highly affected by the weed infestation worldwide. The development of HR crops and application of herbicides have become an economical method for weed management. The shikimate pathway is an important and indispensable pathway in plants, fungi and microbes, and the EPSPS enzyme in this pathway is a primary target for the development of GR crops (Helander *et al*., 2012). Glyphosate is the most widely used systematic broad‐spectrum herbicides and has significantly revolutionized modern agriculture by controlling weeds in crop fields, which has further helped in subsequently increasing global crop productivity (Green, 2014). Based on the U.S. Department of Agriculture (USDA) reports, 94% soybean, 91% cotton and 90% maize cultivated in the United States are herbicide‐tolerant (USDA, 2018). The naturally occurring glyphosate‐insensitive type II Aro enzyme from *Agrobacterium* sp. strain CP4 was the first gene to be used for imparting glyphosate tolerance in many economically and commercially important crop species (Wang *et al*., 2014). Since then, efforts have been made to develop several EPSPS‐tolerant mutant variants by site‐directed mutagenesis. However, not all mutations have led to the development of GR crops. Further, cultivation of transgenic crops has remained a subject of ethical controversy in many parts of the world and has resulted in increased expenditure for the production of transgenic plants, as a substantial amount of resources is being used for devising strategies for avoiding the escape of transgenes into the environment and for addressing public safety concerns (Qaim and Kouser, 2013). Hence, identification of naturally occurring novel glyphosate‐tolerant mutations and introduction of the same in crop‐specific EPSPS genes for the development of glyphosate‐tolerant crops that would enable the practice of sustainable agriculture are urgently needed.

Site‐directed mutagenesis is among the methods for improving the aroA enzyme for glyphosate resistance. However, while introducing the mutations for glyphosate tolerance, the binding affinity for S3P and PEP and, the catalytic efficiency of the enzyme has a chance of being eliminated or reduced due to modifications in the three‐dimensional conformation of the enzyme. Thus, exploring the new combinations of naturally available mutations for glyphosate tolerance in weed species and introducing the same into native aroA enzymes is necessary to achieve field level tolerance against glyphosate, without compromising the affinity of the EPSPS enzyme for PEP or S3P. The glyphosate resistance amino acid substitution mutation P106S was first identified in *E. indica* (Baerson *et al*., 2002). In addition, several other P‐106 substitution mutations to ‐T, ‐A or ‐L have been reported in GR populations of *E. indica* (Ng *et al*., 2003), *L. rigidum* (Wakelin and Preston, 2006) and *L. rigidum* (Yu *et al*., 2007), respectively (Table S2). Similarly, using ethyl methanesulfonate mutagenesis, Comai *et al*. (1983) reported the same P106S mutation (177 in rice) in *S. typhimurium*. From these findings, it can be concluded that the high incidence of the P‐106 substitution mutation to ‐S, ‐T, ‐A or ‐L occurs in the EPSPS enzyme of bacteria and plants, which further indicates that P‐106 might be a common mutation point for glyphosate resistance (Zhou *et al*., 2006). Additionally, overexpression of the P101S (177 in rice) EPSPS mutant from *S. typhimurium* in transgenic tobacco can increase glyphosate tolerance (Comai *et al*., 1985). The *Agrobacterium* sp. CP4 strain is naturally tolerant to glyphosate; this strain has a single A‐100 amino acid substitution that causes the *aroA* gene to become insensitive to glyphosate. The A‐100 residue is present in the active site of the EPSPS enzyme and is highly conserved among plants and bacteria (Funke *et al*., 2006). Glyphosate‐resistant G96A mutations have also been identified in *K. pneumoniae* (Sost and Amrhein, 1990) and *E. coli* (Eschenburg *et al*., 2002). Molecular analyses have revealed that, due to the protrusion of the methyl group of A into the glyphosate‐binding site, glyphosate fails to bind to the active site of the EPSPS enzyme, thus providing protection to the herbicide (Pollegioni *et al*., 2011). Further, EPSPS multisite substitution mutations for high‐level glyphosate resistance with additional complementary properties have been identified in *Petunia hybrida* (GAGD; G101A + G137D and GAPS; G101A + P158S) (Padgette *et al*., 1996), *E. coli* (GAAT; G96A + A183T) (Eichholtz *et al*., 2001; Kahrizi *et al*., 2007) and *E. indica* (TIPS; T102I + P106S) (Yu *et al*., 2015). *Zea mays* (field corn, GA21 event) was the first glyphosate‐tolerant commercial variety harbouring the double mutation (T102I and P106S) to be grown worldwide and contains the only class I enzyme that is commercially used to make the plants insensitive to glyphosate (Ki > 2 mm) without compromising the affinity of PEP towards EPSPS. (Dams *et al*., 1995; Lebrun *et al*., 1997; Lebrun *et al*., 2003). Yield of this maize was not affected by early application of glyphosate (Gower *et al*., 2002; Gower *et al*., 2003; Pline‐Srnic, 2006). Based on the naturally available mutations from the glyphosate‐tolerant weeds, double (T173I + P177S; *TIPS‐OsEPSPS*) and triple (G172A + T173I + P177S; *GATIPS‐OsEPSPS*) amino acid substitution mutations were introduced in the rice *EPSPS* gene by PCR based site‐directed mutagenesis and subsequently used the mutant variants to develop glyphosate‐tolerant rice plants.

The structural analysis of the *E. coli* EPSPS protein revealed that PEP physically interacts with the 17th amino acid of the EPSPS enzyme; hence, any change in the amino acid residue at the PEP binding site prevents glyphosate entry (Schonbrunn *et al*., 2001). The results of the *E. coli* growth inhibition study also support those of previous reports in which the single P106S substitution mutation (corresponding to P177S in rice) resulted in a relatively low level of glyphosate tolerance (Arnaud *et al*., 1998). The amino acid substitution T102I (corresponding to T173I in rice) alone provided strong glyphosate resistance; however, this mutation reduced the catalytic binding affinity of the PEP substrate (Funke *et al*., 2009). However, another study reported that the T to I mutation (T97I) variant provides relatively less resistance against glyphosate, and the presence of the P101S mutation significantly reduces the PEP affinity (Funke *et al*., 2009; Pollegioni *et al*., 2011). The concomitant adjacent mutations at amino acid positions 106 and 102 in the active site of *E. coli* EPSPS (corresponding to T173I and P177S in rice) constitute the only combination of mutations that offers small conformational changes resulting in the nonbinding of glyphosate to the EPSPS enzyme while simultaneously maintaining intact PEP binding efficiency (Funke *et al*., 2009). In support of the above facts, compared with the triple (*GATIPS‐OsEPSPS*) substitution mutation, the double substitution mutation (*TIPS‐OsEPSPS*) provided a higher level (50 mm) of glyphosate tolerance in the DC lines (Figure 5a). Additionally, the overexpression of *OsEPSPS* containing TIPS mutations in plants neutralized the negative effects of glyphosate.

To validate the function of a candidate gene, the use of transgenic platforms is the best approach. In the present study, the *TIPS‐OsEPSPS* and *GATIPS‐OsEPSPS* overexpressing rice lines under different promoters were successfully generated. Attempts have been made to engineer rice plants for glyphosate resistance by overexpressing the aroA gene from different organisms, such as *M. domestica* (Tian *et al*., 2013) and *Janibacter* spp. (Yi *et al*., 2016). The results from the present study showed that, compared with *GATIPS‐OsEPSPS‐*expressing rice lines, *TIPS‐OsEPSPS* rice lines showed high degree of glyphosate tolerance (Figures 4 and 5). Further, much higher tolerance to glyphosate resulted from the constitutive ZmUbi promoter‐regulated overexpression of *TIPS‐OsEPSPS* than from the native EPSPS promoter‐regulated expression (Figures 3 and 4). Promoters are important for driving transgene expression in engineered plants and are essential components for the development of crops that present superior yield and quality as well as a greater degree of tolerance against abiotic and biotic stresses. The success of transgenic plants is determined by both the efficiency of biological function of the overexpressed proteins and the strength of their expression in the overall plant system or desired tissues. Among the constitutive promoters, the CaMV35S viral promoter has been among the most widely used promoters in basic research and in the development of transgenic plants due to the high levels of transgene expression in dicots. However, its expression is relatively less in monocots (Gupta *et al*., 2001; Weeks *et al*., 1993). On the other hand, monocot‐derived promoters including *ZmUbi1*, *OsAct1*, *OsCc1*, *OsAct2* and *OsUbi1* have displayed higher activity in monocots and have been successfully used to develop transgenic crops. The ZmUbi1 promoter is one of the best and is routinely used in the monocot crop species; this promoter causes high levels of gene expression in nearly all tissues at different developmental stages (Park *et al*., 2010). Further, the study also confirmed strong GFP expression in floral tissues including anther, filament and reproductive tissues such as lemma, palea, lodicules, aleurone layer and embryo of rice (Park *et al*., 2010). In the present study, DC lines exhibited enhanced glyphosate tolerance is due to high level of *OsEPSPS* expression driven by strong constitutive ZmUbi promoter. Some of the previous studies involving overexpression of modified EPSPS witnessed reduced pollen viability upon glyphosate treatment. For instance, when rice actin promoter was used to drive expression of modified maize EPSPS (T102I and P106S substitution) in GA21 glyphosate resistance event of corn, the transgenic plants witnessed reduced pollen viability upon glyphosate spray. Similar observation was recorded in the NK603 event of corn which had 35S promoter to drive expression of modified CP4 gene (L214P) (Thomas *et al*., 2004). Similarly, Chen and Hubmeier (2001) reported that reduced pollen viability caused by late glyphosate treatment in glyphosate‐resistant cotton was due to low expression of the 35S promoter‐driven CP4 EPSPS in the male reproductive tissue. Interestingly, we noted that glyphosate application up to 8 mL/L did not show any loss of pollen viability in the DC1 and DC2 transgenic rice lines (Figure S6). This could be due to ZmUbi promoter‐driven strong expression of the modified EPSPS in the reproductive tissues of rice which was also previously demonstrated using GFP (Park *et al*., 2010).

With the recent introduction of genome editing technologies, accurate base alterations in the genome of crop plants have become possible offering great potential for crop improvement. CRISPR/Cas9 editing system has been successfully used to develop non‐GM herbicide‐resistant crop plants, targeting both *EPSPS* and *acetolactate synthase* (*ALS*) genes against most widely used herbicides (Zhang *et al*., 2018). The TIPS mutation has been introduced in rice through gene replacement using CRISPR‐Cas9 technology (Li *et al*., 2016). The study showed that newly developed TIPS introduced plants could tolerate up to 400X diluted glyphosate solution (Roundup with 41.0% glyphosate) (Li *et al*., 2016). Similarly, genome editing technology has also been used to introgress glyphosate tolerance in flax and cassava through allele exchange in the EPSPS locus (Hummel *et al*., 2018; Sauer *et al*., 2016). The gene‐editing technology can introduce desired mutations in the EPSP synthase to make it glyphosate tolerant; however, a strong constitutive promoter system is also essential to increase the expression of the modified EPSPS gene for achieving field level tolerance. Li *et al*. (2016) could achieve a very limited level of glyphosate tolerance by introducing TIPS mutation in *EPSPS* gene of rice (i.e. the edited rice plants could tolerate only 400X (equivalent to 5.25 mm of glyphosate). On the other hand, in the present case, overexpression of TIPS‐*EPSPS* under the control of constitutive *ZmUbi* promoter resulted in development of transgenic rice plants which could tolerate up to 125X diluted commercial Roundup Ready glyphosate herbicide (equivalent to 16.8 mm of glyphosate). The above fact is also in agreement with the present findings which shows that upon expressing *TIPS‐OsEPSPS* under the regulation of *ZmUbi* promoter, the transgenic lines (i.e. DC1 plants) could tolerate higher level of glyphosate than the transgenic lines expressing *TIPS‐OsEPSPS* under its native promoter (i.e. DN4 line). Further, 1000 µm glyphosate treatment completely inhibited root formation in DN4 transgenic line and suppressed its shoot growth (Figure 3a). Thus, the study highlights that rice EPSPS promoter is not sufficient to impart field level glyphosate tolerance in the transgenic plants even after introduction of an extra copy of glyphosate‐tolerant *TIPS* mutation in the rice EPSPS gene. In conclusion, even if TIPS mutation is introduced in rice EPSPS gene using genome editing approach, the edited plants might not be agronomically fit to tolerate high dose of glyphosate normally applied under the field conditions to completely suppress the weeds. From the present study, it may be inferred that transgenic technology with proper combination of the targeted gene and promoter gives a viable solution to achieve field level glyphosate tolerance in rice.

Rice is sensitive to glyphosate causing, severe injury to plants. Our results revealed that, compared with WT lines, DC lines had a greater degree of tolerance to different doses of glyphosate, maximum up to 21 mm in pot conditions and 16.8 mm in field condition (Figure 5a and 6a) without any productive fitness cost; the WT plants were died 7 days after the glyphosate treatment at dose of 4.2 mm. The present tolerance level is comparable to that in previous reports involving transgenic overexpression of several class II *aroA* genes in tobacco (Yan *et al*., 2011), rice (Chhapekar *et al*., 2015; Yi *et al*., 2015), *Arabidopsis* (Tian *et al*., 2012) and maize (Ren *et al*., 2015). During the present study, it was also observed that the application of glyphosate resulted in the death of monocot (WT rice, *C. barbata*) and dicot (*P. niruri*, *E. hirta*) weeds in the crop–weed competition experiment (Figure 5b). The DC lines treated with glyphosate displayed normal physiology, and their yields were relatively enhanced to those of untreated WT plants under normal field conditions (Figure 6; Table 1a).

The growth and yield performance of selected lines (DC and TC) were studied under controlled conditions (without glyphosate application). Interestingly, the DC1 and DC2 lines produced 17‐ 19% more grain yields as compared to the WT control plants (Table 1b). The improvement in yield of the DC plants is attributed to increase in the total number of seeds per plant as well as seeds per panicle (Table 1b). The other yield parameters, namely number of tillers per plant, individual seed weight and 100 grain weights of the DC plants did not change significantly in comparison with the WT plants. The shikimic acid pathway is an indispensable biochemical pathway in plants that provides precursors for the biosynthesis of chorismate, aromatic amino acids and other aromatic secondary metabolites required for plant growth and development. Chorismate is one of the central branch point from where many metabolites including precursor for vitamins tetrahydrofolate (vitamin B9) and phylloquinone (vitamin K1) and the plant defence hormone salicylate are produced (Maeda and Dudareva, 2012). Similarly, phenylalanine is the precursor molecule of many secondary phenylpropanoids including volatiles, glucosinolates, flavonols, flavones, isoflavanones, isoflavones, anthocyanin and tannins. Our study revealed about 6.5‐fold increase in the phenylalanine level in the DC transgenic seeds as compared to the wild‐type seeds. Phenylalanine is found in many protein‐containing foods, and it is important for normal functioning of human body. The amount of this amino acid found in foods should not pose a risk for otherwise healthy individuals and is ‘generally recognized as safe’ by the Food and Drug Administration (FDA). No side effects are generally observed at supplement doses of 23–45 mg per pound (50–100 mg per kg) of body weight. Moreover, high level of phenylalanine is required to cure skin disorders such as vitiligo and alleviate the depression problems due to dopamine malfunction (Cohen *et al*., 2015; Meyers, 2000). Brown rice contains about 133 mg/100g of phenylalanine (Kalman, 2014). In the present research, phenylalanine content of transgenic rice has been found to increase by 6.5‐fold thus making the phenylalanine level up to 865 mg/100 g (6.5 times of 133 mg/g). This increased level of phenylalanine falls within the safe limit of phenylalanine content of foods, and it is well below the phenylalanine content of soy protein (3278 mg/100 g; Kalman, 2014) which is normally consumed by people. Thus, the amount of phenylalanine present in the transgenic rice grain is negligible to cause any health hazards. However, the individuals with metabolism disorder of phenylketonuria (PKU) are advisable to be placed on a special low‐protein diet for life.

In plants, about 30% of photosynthetic carbon is incorporated for the biosynthesis of phenylpropanoids compounds (Maeda and Dudareva, 2012). The other essential aromatic secondary metabolites including plastoquinones, tocochromanols (vitamin E), non‐protein amino acids and isoquinoline alkaloids are derived from the tyrosine source. Plant tocopherols are antioxidants which provide protection to chloroplastic membrane against photo‐oxidation injury. They also protect polyunsaturated fatty acids and hunting oxyfree radicals produced under different environmental conditions (Collakova and DellaPenna, 2003; Munne‐Bosch and Alegre, 2002). Similarly, tryptophan derived secondary substances such as glucosinolates, an essential compound involved in the plant–pathogen and plant–insect interactions and camalexin a major indolic phytoalexin is also produced *via* this pathway, which are implicated in many biotic and abiotic elicitations (Ahuja *et al*., 2012). The master signalling growth regulator indole‐3‐acetic acid (IAA, auxin) is synthesized from tryptophan, which is essential for plant growth and development. The DC transgenic seeds were observed to have about twofold increase in the tryptophan level during the study as compared to the WT seeds. The above facts support the observations made during the study that the DC lines gave higher grain yield compared to the wild type. The overexpression of the crucial enzyme of shikimate pathway, that is EPSP synthase can lead to more fitness advantages to rice plants which in turn results in increased productivity and yield. A similar finding was also reported in the rice earlier where the transgenic overexpression of modified EPSPS showed more fitness advantages over the non‐transgenic WT plant with increased EPSPS protein and tryptophan level, and high photosynthetic and seed germination rates (Wang *et al*., 2013). Another study by Wang *et al*. (2013) identified that overexpression of a novel EPSPS transgene for glyphosate resistance increased growth and fecundity in weedy rice in absence of glyphosate treatment. However, these fitness advantage traits due to glyphosate‐insensitive EPSPS overexpression can easily spread to weedy rice species that can cross‐pollinate with the crop plants (Arrigo *et al*., 2011; Ellstrand, 2003; Perez‐Jones *et al*., 2006; Rehman *et al*., 2006; Wang *et al*., 2013; Weissmann *et al*., 2005). Recent evidences show that most of the weedy rice species have arisen from de‐domestication of cultivated rice varieties and not from the common wild progenitors (Gross *et al*., 2010; Lawton‐Rauh and Burgos, 2010; Thurber *et al*., 2013). Thus, increase in the fitness and fecundity of weedy rice strains is due to introgression of genes from cultivated crop plants those have been continuously selected for increased yield traits since the onset of agriculture (Gressel *et al*., 2014). Nevertheless, whenever herbicide‐tolerant transgenic crops are being grown, there are chances of evolution of super weeds and gene flow of herbicide resistance from herbicide‐tolerant rice to weedy rice is rapid once intraspecific hybrids form among the aforesaid rice plants (Gealy *et al*., 2003). But, there are multiple ways to prevent the flow of herbicide‐tolerant transgene from cultivated to weedy plants. Physical containment methods (utilizing filters in laboratories, mesh on greenhouses) and biological containment methods (such as growing cross‐pollinated plants at different time points so that their flowering time do not coincide, single generation transformation using disarmed viruses, employing male sterility to prevent gene transfer through pollen, genetic use restriction technology (GURT) or terminator technology) are some of the ways to contain transgene flow to weedy crop species (Gressel, 2015). More precise mitigation technologies such as use of mitigator genes (e.g. genes for dwarfing or no seed shatter or floral abortion or no bolting or preventor of homologous recombination or delayed flowering or the use of a highly specific zinc‐finger nuclease that would target weed’s genes encoding seed shattering resistance) have been employed to prevent gene flow to the weedy species of crop plants. In these cases, even if the transgene flows into its weedy crop relatives, the mitigator genes which are also transferred along with the transgene would impart negative characteristics to weed which will in turn prove deleterious for perpetuation of weeds under crop–weed competition in fields (Gressel, 2015). Other strategies including the development of transgenic crops that are tolerant to two or more broad‐spectrum herbicides are recommended which constitutes an effective integrated weed management practice to counter the development of super weeds in the agriculture (Fartyal *et al*., 2018). Though the use of multiple herbicides in rotation for weed control do not prevent transgene flow, their alternate use poses less selection pressure on weeds resulting in infrequent development of super weeds. Moreover, such rotations would incessantly kill weed × crop hybrids as well as any evolved herbicide‐resistant weeds, thus dealing with the problems of transgene flow and development of super weeds. Further, various other strategies have been employed for developing glyphosate‐tolerant plants by utilizing either glyphosate‐tolerant EPSPS gene variants alone or in combination with glyphosate detoxifying genes. Among them, use of bacterial glyphosate detoxifying genes such as *gat* (glyphosate N‐acetyl transferase) (Castle *et al*., 2004; Delaney *et al*., 2008; Dun *et al*., 2014; Guo *et al*., 2015; Liu *et al*., 2015) and *gox* (glyphosate oxidoreductase) (Howe *et al*., 2002) is prominent. The gox gene was used to provide glyphosate resistance in canola and maize in combination with CP4 EPSPS gene (Barry and Kishore, 1995). Zhou and colleagues developed glyphosate‐tolerant wheat transgenic plants by transforming both CP4 EPSPS and gox genes together and also utilized glyphosate as a selection marker to develop the transgenic plants using 2 mm glyphosate (Zhou *et al*., 1995). Transgenic canola plants expressing codon‐optimized gox was developed and these plants could tolerate 1 mm of glyphosate spray (Hadi *et al*., 2012). Another glyphosate detoxifying enzyme, glyphosate N‐acetyltransferase (GAT) particularly from the *Bacillus licheniformis* strains is worthy of discussion. Castle *et al*. (2004) screened a wide array of *Bacillus* species and found that GAT proteins from *Bacillus licheniformis* strains were catalytically most active with K_m_^glyphosate^ of 1.2 to 1.8 mm and K_cat_ of 1.0 to 1.7 min^−1^. However, the same genes could not impart glyphosate tolerance in transgenic tobacco and *Arabidopsis*. Therefore, the group used *gat* genes from three strains (namely ST401, B6 and DS3) of *Bacillus* for carrying out fragmentation‐based multigene shuffling with the aim of creating enzymes with higher efficiency and increased glyphosate specificity for developing glyphosate‐tolerant transgenic plants. The most efficient GAT variant developed had K_m_ of 0.05 mm and K_cat_ of 416 min^−1^. This gene when overexpressed in maize imparted high level of glyphosate tolerance (Castle *et al*., 2004). Other glyphosate metabolizing genes such as D‐amino acid oxidase (DAAO) (Han *et al*., 2015), glycine oxidase (GO) (Nicolia *et al*., 2014) and increased glyphosate resistance (igrA) (Fartyal *et al*., 2018; Vemanna *et al*., 2017) have also been characterized and used for developing glyphosate‐tolerant transgenic plants. These genes metabolize glyphosate into lesser toxic compounds and impart glyphosate tolerance to the transgenic plants. In the present research, the crop‐specific constitutive overexpression of TIPS mutation in EPSPS gene provides higher degree of glyphosate tolerance along with effective controlling of all kinds of prevailing weeds with glyphosate application and it can be hugely emulated in thecrop improvement programme for transferring the elite traits into the popular cultivars for weed management and improved productivity (Figure 7b). In future, suitable mitigation technology along with enzymatic detoxification of glyphosate can be combined with the present method of weed control to further improve glyphosate tolerance and mitigate transgene flow into the weedy plant species of rice.

Multiple mutations in a single gene against a particular herbicide confer greater advantage to a plant in adapting to the particular herbicide and hence are the main cause of evolution of super weeds (Brunner *et al*., 2008; Karasov *et al*., 2010). Naturally, in majority of the cases, evolution of multiple point mutations in a single allele occurs *via* recombination between natural plant populations harbouring single point mutations (Brunner *et al*., 2008; Mutero *et al*., 1994). Adoption of high glyphosate‐tolerant plants containing TIPS mutation in the *EPSPS* gene in the modern agriculture will be advantageous as it will facilitate application of high dose of glyphosate under field conditions for controlling all the weed species. Higher dose of glyphosate spraying will not only combat the weeds efficiently but also will prevent evolution of weeds containing single glyphosate‐tolerant point mutations as single point mutations are seen to confer lower resistance to glyphosate. Hence, cultivation of TIPS mutation containing rice can be a part of sustainable agriculture for vast majority of Asian and African countries where rice is the staple crop and is widely cultivated.

## Experimental procedures

All experimental procedures involving chemicals and materials, sequence analysis and bioinformatic study, experimental design, site‐directed mutagenesis, expression cassettes details, molecular, biochemical and physiological analysis of transgenic plants and data analysis are available in the online version of the journal (Experimental procedures S1).

## Conflict of interest

The authors declare no conflict of interest.

## Author contributions

VMMA carried out the experiments including site‐directed mutagenesis, cloning, molecular confirmation, physiological analysis, plant tissue culture, data analysis and drafted the manuscript. VMMA and MM performed expression analysis, in vitro physiological experiments and drafted the revised version of the manuscript. VMMA, VP and DF were involved in making the expression cassettes. VS, VMMA, AA, BR and BB generated overexpressed rice plants. SKM and DT carried out GC‐MS analysis. MKR and PKA helped in planning the research, discussion during analysis and finalization of the manuscript.

## Supporting information


**Experimental Procedures.**
**Figure S1.** (a) Polynucleotide sequence of rice EPSPS promoter and (b) rice EPSPS terminator. Highlighted nucleotide sequence represents the primer sequence along with restriction sites.
**Figure S2.** (a) Polynucleotide sequence of Zea mays polyubiquitin 1 (ZmUbi) promoter and (b) nopaline synthase gene terminator. Highlighted nucleotide sequence represents the primer sequence along with restriction sites.
**Figure S3.** Polynucleotide DNA sequence of rice EPSP synthase. The blue colour text represent chloroplast transit peptide sequence.
**Figure S4.** Polynucleotide DNA sequence of mutant (T/173/I and P/177/S) TIPS‐OsEPSPS. The blue colour text represents chloroplast transit peptide sequence. The amino acid substitution mutations T/173/I and P/177/S are highlighted in pink and yellow colour respectively.
**Figure S5.** Polynucleotide DNA sequence of mutant (G/172/A, T/173/I and P/177/S) GATIPS‐OsEPSPS. The blue colour text represents chloroplast transit peptide sequence. The amino acid substitution mutations G/172/A, T/173/I and P/177/S are highlighted in red, pink and yellow colour respectively.
**Figure S6.** Pollen viability test. The viable pollen grains from untreated WT and glyphosate treated DC1 and DC2 transgenic plants stained by 2 % aceto‐carmine. The bars represent 50 µm.
**Figure S7.** Relative levels of free aromatic amino acids in rice seeds extracted in Aqueous methanol chloroform (MeOH: CHCl_3_: H_2_O (5:2:1)) and analysed by GC‐MS after TBDMS derivatisation. The relative peak abundances of Phenylalanine, Tyrosine and Tryptophan in wild type (WT) and transgenic rice seeds were normalised to L‐norleucine (60 µL of 0.2 mg/mL) as internal standard with abundance set at 100.
**Table S1.** List of primers used in the study
**Table S2.** Glyphosate resistance amino acid substitutions mutations (in EPSPS) identified in resistance‐weed biotypes
**Table S3.** Similarity percentage of protein sequences among various EPSPS enzymes from different organisms.
