# Supplementary material for: Overexpression of improved EPSPS gene results in field level glyphosate tolerance and higher grain yield in rice
Source: Plant Biotechnol J. 2020 Jul 24;18(12):2504–19. doi: 10.1111/pbi.13428 (PMC7680544; doi:10.1111/pbi.13428)
Supplement: Supplementary file 1 — Experimental Procedures. Figure S1. (a) Polynucleotide sequence of rice EPSPS promoter and (b) rice EPSPS terminator. Highlighted nucleotide sequence represents the primer sequence along with restriction sites. Figure S2. (a) Polynucleotide sequence of Zea mays polyubiquitin 1 (ZmUbi) promoter and (b) nopaline synthase gene terminator. Highlighted nucleotide sequence represents the primer sequence along with restriction sites. Figure S3. Polynucleotide DNA sequence of rice EPSP synthase. The blue colour text represent chloroplast transit peptide sequence. Figure S4. Polynucleotide DNA sequence of mutant (T/173/I and P/177/S) TIPS‐OsEPSPS. The blue colour text represents chloroplast transit peptide sequence. The amino acid substitution mutations T/173/I and P/177/S are highlighted in pink and yellow colour respectively. Figure S5. Polynucleotide DNA sequence of mutant (G/172/A, T/173/I and P/177/S) GATIPS‐OsEPSPS. The blue colour text represents chloroplast transit peptide sequence. The amino acid substitution mutations G/172/A, T/173/I and P/177/S are highlighted in red, pink and yellow colour respectively. Figure S6. Pollen viability test. The viable pollen grains from untreated WT and glyphosate treated DC1 and DC2 transgenic plants stained by 2 % aceto‐carmine. The bars represent 50 µm. Figure S7. Relative levels of free aromatic amino acids in rice seeds extracted in Aqueous methanol chloroform (MeOH: CHCl3: H2O (5:2:1)) and analysed by GC‐MS after TBDMS derivatisation. The relative peak abundances of Phenylalanine, Tyrosine and Tryptophan in wild type (WT) and transgenic rice seeds were normalised to L‐norleucine (60 µL of 0.2 mg/mL) as internal standard with abundance set at 100. Table S1. List of primers used in the study Table S2. Glyphosate resistance amino acid substitutions mutations (in EPSPS) identified in resistance‐weed biotypes Table S3. Similarity percentage of protein sequences among various EPSPS enzymes from different organisms. [file PBI-18-2504-s001.zip › pbi13428-sup-0001-Experimental Procedures (S1).docx]

**EXPERIMENTAL PROCEDURES**

**Chemicals and materials**

Most of the chemicals were purchased from Sigma Chemical Corporation, Ltd. (St. Louis, MO). Primers used in this study were synthesized by Integrated DNA Technologies (Leuven, Belgium). Blotting kits were purchased from Roche Diagnostics GmbH. (Germany), and LR Clonase, entry vectors and pMDC99 vectors were purchased from Thermo Fisher Scientific Corporation, USA. Restriction enzymes were obtained from New England Biolabs, MA. The rice cultivar Japonica (variety Nipponbare) was used for the transgene validation.

**Sequence analysis of rice EPSPS**

The nucleotide and amino acid sequences of EPSPS were analyzed and compared using the BLAST program (NCBI, http://www.ncbi.nlm.nih.gov). Phylogenetic analyses of EPSPS orthologs from different organisms were performed using the ClustalW program (www2.ebi.ac.uk/clustalw), and a phylogenetic tree was constructed by the neighbor-joining method *via* MEGA version 6.0. A molecular model of EPSPS was generated using the Swiss Model database (Arnold, 2006); the crystal structure of the *Vibrio cholerae* protein (Protein databank ID 3nvs.1) served as template. The PyMOL program (http://pymol.sourceforge.net/) was used to depict the EPSPS molecular model structure.

**Isolation of the rice EPSPS gene and site-directed mutagenesis**

Total RNA was isolated from two-week-old rice plants following TRI-ZOL™ method (Life Technologies). Two micrograms of mRNA was used as a template for first-strand cDNA synthesis following the protocol of the Verso cDNA synthesis kit (Thermo Scientific, USA). The full-length coding DNA sequence (CDS) of the rice *EPSPS* gene (*OsEPSPS*) including the chloroplast transit peptide (1548 bp; Figure S3) was amplified using *EPSPS* gene-specific forward (5′-ATGGCGGCGACCATGGCGTC-3′) and reverse (5′- TCAGTTCCTGACGAAAGTGCTTAGA-3′) primers. The amino acid substitutions glycine (G) to A, T to isoleucine (I) and P to S at amino acid positions 172, 173 and 177 respectively, in the active site region of *OsEPSPS* was carried out *via* polymerase chain reaction (PCR). For the P to S (P177S) mutation, the codon CCA was replaced with AGC. For the T to I (T173I) mutation, the codon was changed from ACT to ATT, and the codon GGA was changed to GCA for the G to A (G172A) mutation. The altered codon sequences were part of the reverse primer sequence to introduce the desired mutations by PCR, and a unique *Hind*III restriction site (AAGCTT) was also introduced in the mutated gene sequence of EPSPS *via* modulation of the base pair sequences of the overlapping primers (Table S1). Furthermore, the DNA fragments (1-540 and 520-1548 bp) were amplified separately and joined *via* *Hind*III digestion and ligation to create double (*TIPS-OsEPSPS*; Figure S4) and triple (*GATIPS-OsEPSPS*, Figure S5) amino acid substitution mutations in the *EPSPS* of rice (Figure 2a). The *Hind*III restriction enzyme was further used as a marker to differentiate WT plants from overexpressed ones.

**Construction of expression cassettes and plant transformation**

To make rice plant more robust to higher dose of glyphosate application, different expression cassettes of *TIPS-OsEPSPS* and *GATIPS-OsEPSPS* under the regulation of ZmUbi constitutive promoter and the native *OsEPSPS* gene promoter (OsEPSPS-P) were generated. The constitutive expression of the *OsEPSPS* cassettes (ZmUbi-P: *TIPS-OsEPSPS*: nos-T and ZmUbi-P: *GATIPS-OsEPSPS*: nos-T) was made *via* the regulation of the ZmUbi promoter and *nosT* (Figure S2)*.* Similarly, the native expression cassettes (OsEPSPS-P: *TIPS-OsEPSPS*: OsEPSPS-T and OsEPSPS-P: *GATIPS-OsEPSPS*: OsEPSPS-T) were made using -1036 bp upstream region of the *OsEPSPS* gene promoter, and its corresponding 236 bp 3’ regulatory terminator sequence (Figure S1). All the expression cassette components were initially cloned into EV-pL12R34-Amp entry vector (Figure 2b), which were subsequently cloned into the Gateway-compatible plant transformation vector pMDC99 *via* a Gateway cloning system (Curtis & Grossniklaus, 2003).

Two-week-old *Oryza sativa* (cultivar Japonica, variety Nipponbare) calli were transformed with *A. tumefaciens* (EHA105) harboring mutant *OsEPSPS* expression cassettes, and cocultivation was performed for 48 h at 24 °C in the dark. The transformed calli were selected on Chu’s (N6) media supplemented with 50 mg/L hygromycin. After three rounds of selection, the calli were transferred to pre-regeneration media for 30 days, after which they were transferred to regeneration media for approximately one month for shoot development. The selected shoots were subsequently transferred to half-strength Murashige and Skoog (MS) rooting media supplemented with 50 mg/L hygromycin for 15 days. After the rice seedlings had completely rooted, they were transplanted in the greenhouse.

**Identification and expression analysis of events generated**

Rice seeds from WT plants and T1 lines were germinated in half-strength Chu’s (N6) media supplemented without and with hygromycin (30 mg/L) for ~10 days, after which the seedlings were transferred to pots containing soil. For transgene identification, genomic DNA was isolated from 21-day-old WT and overexpressed lines and confirmed by PCR *via* gene-specific primers (Table S1). To determine the copy numbers of the transgene(s), Southern blot analyses were carried out. Approximately 15 μg of genomic DNA from the WT and overexpressed lines were digested with 100 units of the *EcoR* V restriction enzyme for 20 h. The digested products were size-fractionated on a 0.8% agarose gel and subsequently transferred onto N^+^ nylon membranes. The blots were initially hybridized with digoxigenin (DIG)-labeled *hpt+* and subsequently re-hybridized with *OsEPSPS* probes respectively. Hybridization and post-hybridization washing conditions and detection of signals were performed in accordance with the instructions provided with the kit (Roche).

For the Northern blot analyses, total RNA from the selected 30-day-old overexpressed T2 lines and their corresponding non-transformed control plants was isolated using Trizol reagent. The total RNA (15 μg) from each overexpressed line was electrophoresed on denaturing agarose gel containing formaldehyde and then transferred to an N+ nylon membrane. The blots were hybridized with the EPSPS coding region (546 bp) as a probe, which was prepared with a PCR DIG probe synthesis kit (Roche). Membrane washing and detection procedures were performed in accordance with the manufacturer’s instructions (Roche).

In order to monitor the expression of *Os-mEPSPS* transcripts, reverse transcription-PCR (RT-PCR) was performed with specific primers, namely, EPSPS-F and EPSPS-R (Table S1); the cDNA from the WT and overexpressed lines served as a template. To differentiate between the PCR-amplified *OsEPSPS* fragments from the native and overexpressed plants, the amplified DNA fragments were digested with *Hind*III restriction enzymes. A unique *Hind*III restriction site was created while making the clones (*TIPS-OsEPSPS* and *GATIPS-OsEPSPS*) of the *OsEPSPS* gene; the relevant details are described in the section ‘Isolation and site-directed mutagenesis in *OsEPSPS*’.

**Validation of the modified plants for resistance to glyphosate**

Overexpressed rice plants harboring the *TIPS-OsEPSPS* gene under the regulation of the constitutive promoter were designated DC lines, and those containing the *TIPS-OsEPSPS* gene under the regulation of the native promoter were designated DN lines. Further, overexpressed rice plants containing the *GATIPS-OsEPSPS* gene under the regulation of the constitutive promoter were designated TC lines, and those containing the *GATIPS-OsEPSPS* gene under the regulation of the native promoter were designated TN lines. Rice seeds from the WT and T2 homozygous overexpressed lines were germinated on half-strength MS media (2.2 g/l MS, 15 g/L sucrose, and 4 g/L gelrite at a final pH of 5.8) supplemented with and without hygromycin (30 mg/L) for 10 days, after which the seedlings were transplanted to soil pots. For short-term pot experiments, rice seedlings were grown in a growth chamber, but for full-season crop, up to maturity, the plants were grown in a greenhouse under natural light or natural light supplemented with light using high-pressure sodium bulbs (50 mM/m^2^/s). Both the growth conditions were with a cycle of 12 h light (30 °C)/12 h dark (28 °C) photoperiod, a photosynthetic photon flux density of approximately 200 μM/m^2^/s and a relative humidity of 70%.

In order confirm the effects of glyphosate on the seedling morphology of rice, seed from both WT and different overexpressed rice lines were germinated on half-strength MS media supplemented with glyphosate (100 µM) in magenta boxes under controlled environmental conditions (25 °C, 10:14 h light/dark photoperiod). The effects of glyphosate on rice seedling morphology was recorded at 21 days after seed germination. To confirm the level of glyphosate tolerance, the DC and DN seeds were grown half strength MS media supplemented with 1000 µM glyphosate. Similarly, the level of glyphosate tolerance by DC1 and TC3 line were also checked using range of glyphosate concentrations (0.1-50 mM), supplemented in the half-strength germination media.

One-month-old T2 seedlings were sprayed with different doses including 2 mL/L (4.2mM), 4 mL/L (8.4mM), 6 mL/L (12.6mM), 8 mL/L (16.8mM) and 10 mL/L (21mM) commercial glyphosate (containing 41.0% glyphosate isopropylamine salt [equivalent to 356gm/L of glyphosate] Monsanto Inc., Montreal, QC, Canada) under controlled greenhouse conditions. The experiment was carried out to see the effect of glyphosate on plant morphology under the greenhouse condition.

We conducted crop-weed competition experiments and mimicked rice fields by growing WT, T2 overexpressed lines along with agronomically important weed species, namely, *Phyllanthus niruri*, *Euphorbia hirta* and *Chloris barbata* in the treatment tray. Glyphosate dose 3mL/L (6.3mM) was foliar sprayed on the weeds and rice lines (WT, DC1 and DC2) and symptoms were recorded in different-day intervals.

The growth and yield penalty of the overexpressed lines in the glyphosate treated field conditions were also assessed. Twenty-day-old seedlings from the WT and T3 overexpressed rice lines (DC1 and DC2) were sprayed with commercial glyphosate solution 2 mL/L (4.2mM), 4mL/L (8.4mM), and 8mL/L (16.8mM), and the plants were subsequently allowed to grow until maturity stage. At the maturity stage, yield parameters such as plant height, number of panicles per plant, panicle length and yield per plant were recorded.

**Pollen viability test**

The pollen viability test was carried out using 2% aceto-carmine solution as described by Rathod *et al.*, 2018. One-month old rice seedlings from transgenic lines (DC1 and DC2) were sprayed with the highest dose of commercial glyphosate 8mL/L (16.8mM), and the pollen were collected for checking their viability. The pollens from WT plants without glyphosate treatment were used as positive control.

**Quantification of phenylalanine, tyrosine and tryptophan by GC-MS**

*Extraction procedure****:*** 250 mg samples were extracted in 2 ml cold MeOH: CHCl_3_: H_2_O (5:2:1) with L-norleucine (60 µl of 0.2 mg/ml) as Internal standard. The extract was vortexed vigorously and centrifuged at 14000 rpm/15 min/4°C. The supernatant was mixed with 500 µl H_2_O and 200 µl CHCl_3_ followed by vortexing and centrifuging at 14000 rpm/ 5 min/ 4°C for phase separation. The upper layer was taken out leaving transition phase and acetonitrile was added to it (at the rate of 2.5 times of the upper layer volume) to deproteinize the sample following which the sample was allowed to vacuum dry. To remove any moisture contamination, 50µl dichloromethane was added and the samples were further allowed to dry.

*Tert-butyldimethylsilyl (TBDMS) derivatization:* The dried samples were N-Methyl-N-(tert-butyldimethylsilyl) trifluoroacetamide (MTBDMSTFA) derivatized. For this, 30 µl of pyridine was added to dried sample and incubated at 37°C at 900 rpm for 30 mins. Then 50 ul of MTBSTFA (N-tert-Butyldimethylsilyl-N-methyltrifluoroacetamide) with 1% of t-BDMCS (tert-Butyldimethylchlorosilane) was added and incubated at 60°C at 900 rpm for 1 hr. Samples were centrifuged at 14000 rpm/ 10 min followed by Injecting it for GC-MS analysis.

*GC-MS analysis:* Agilent GC-MS with Mass selective detector (MSD) was used for GC-MS analysis. MassHunter (Agilent Technologies, USA) was used to control the data acquisition parameters (both GC separation and mass spectrometry) during all the sample runs. The intensity of the mass ions of each amino acid fragment was obtained by using Agilent Chemstation software. The gradient was 120°C hold for 5 min, increasing to 270°C at the ramp rate of 4°C/min with holding it at 27 °C for 3 min. Then increasing from 270°C to 320°C at the ramp rate of 20°C/min with holding it at 320°C for 1 min. Injection volume was 1 µl.

**Statistical Analysis**

The quantitative experiments were performed in duplicate or triplicate with biological replications (n) indicated in their respective figures. Pooled data were statistically analyzed for analysis of variance (ANOVA) followed by least significant difference (LSD) at significance level *p* ≤ 0.05 or 0.01.
