# Supplementary material for: Overexpression of improved EPSPS gene results in field level glyphosate tolerance and higher grain yield in rice
Source: Plant Biotechnol J. 2020 Jul 24;18(12):2504–19. doi: 10.1111/pbi.13428 (PMC7680544; doi:10.1111/pbi.13428)
Supplement: Supplementary file 1 — Experimental Procedures. Figure S1. (a) Polynucleotide sequence of rice EPSPS promoter and (b) rice EPSPS terminator. Highlighted nucleotide sequence represents the primer sequence along with restriction sites. Figure S2. (a) Polynucleotide sequence of Zea mays polyubiquitin 1 (ZmUbi) promoter and (b) nopaline synthase gene terminator. Highlighted nucleotide sequence represents the primer sequence along with restriction sites. Figure S3. Polynucleotide DNA sequence of rice EPSP synthase. The blue colour text represent chloroplast transit peptide sequence. Figure S4. Polynucleotide DNA sequence of mutant (T/173/I and P/177/S) TIPS‐OsEPSPS. The blue colour text represents chloroplast transit peptide sequence. The amino acid substitution mutations T/173/I and P/177/S are highlighted in pink and yellow colour respectively. Figure S5. Polynucleotide DNA sequence of mutant (G/172/A, T/173/I and P/177/S) GATIPS‐OsEPSPS. The blue colour text represents chloroplast transit peptide sequence. The amino acid substitution mutations G/172/A, T/173/I and P/177/S are highlighted in red, pink and yellow colour respectively. Figure S6. Pollen viability test. The viable pollen grains from untreated WT and glyphosate treated DC1 and DC2 transgenic plants stained by 2 % aceto‐carmine. The bars represent 50 µm. Figure S7. Relative levels of free aromatic amino acids in rice seeds extracted in Aqueous methanol chloroform (MeOH: CHCl3: H2O (5:2:1)) and analysed by GC‐MS after TBDMS derivatisation. The relative peak abundances of Phenylalanine, Tyrosine and Tryptophan in wild type (WT) and transgenic rice seeds were normalised to L‐norleucine (60 µL of 0.2 mg/mL) as internal standard with abundance set at 100. Table S1. List of primers used in the study Table S2. Glyphosate resistance amino acid substitutions mutations (in EPSPS) identified in resistance‐weed biotypes Table S3. Similarity percentage of protein sequences among various EPSPS enzymes from different organisms. [file PBI-18-2504-s001.zip › pbi13428-sup-0010-TableS2.docx]

**Table S2** Glyphosate resistance amino acid substitutions mutations (in EPSPS) identified in resistance-weed biotypes.

| **Amino acid substitution mutation in EPSPS** | **Plant species** | **Reference** |
| --- | --- | --- |
| Threonine 102 Isoleucine | *Eleusine indica* (Goose grass) | Yu *et al*., 2015 |
| Threonine 102 Serine | *Tridax procumbens* (Coat buttons) | Li *et al*., 2018 |
| Proline 106 to Serine | *Eleusine indica* (Goose grass) | Baerson *et al*., 2002 |
|  | *Lolium perenne ssp. Multiflorum* (Italian ryegrass) | Perez-Jones *et al*., 2007 |
|  | *Lolium rigidum* (Rigid ryegrass) | Simarmata *et al*., 2008 |
|  | *Amaranthus tuberculatus* (Tall Waterhemp**)** | Nandula *et al*., 2013 |
|  | *Echinochloa colona* (Junglerice**)** | Alarcon-Reverte *et al*., 2013 |
| Proline 106 to Threonine | *Eleusine indica* (Goose grass) | Ng *et al*., 2003 |
|  | *Lolium rigidum* (Rigid ryegrass) | Wakelin *et al*., 2006 |
|  | *Digitaria insularis* (Sourgrass) | de Carvalho *et al*., 2012 |
|  | *Echinochloa colona* (Junglerice**)** | Alarcón-Reverte *et al*., 2014 |
| Proline 106 to Alanine | *Lolium rigidum* (Rigid ryegrass) | Yu *et al*., 2007 |
|  | *Lolium perenne ssp. Multiflorum* (Italian ryegrass) | Jasieniuk et al., 2008 |
| Proline 106 to Leucine | *Lolium rigidum* (Rigid ryegrass) | Kaundun *et al*., 2011 |
|  | *Echinochloa colona* (Junglerice**)** | Han *et al*., 2016 |
|  | *Eleusine indica* (Goose grass) | Chen *et al*., 2015 |

Alarcon-Reverte R, García A, Urzúa J, Fischer AJ (2013) Resistance to glyphosate in jungle rice (*Echinochloa colona*) from California. *Weed Science* **61**: 48–54.

Alarcón-Reverte R, García A, Watson SB, Abdallah I, Sabaté S, Hernández MJ, Dayan FE, Fischer AJ (2014) Concerted action of target-site mutations and high EPSPS activity in glyphosate-resistant jungle rice (*Echinochloa colona*) from California. *Pest Management Science* **71**:996–1007.

Baerson SR, Rodriguez DJ, Tran M, Feng YM, Biest NA, Dill GM (2002) Glyphosate-resistant goosegrass. Identification of a mutation in the target enzyme 5-enolpyruvylshikimate-3-phosphate synthase. *Plant Physiology* **129**: 1265–1275.

Chen J, Huang H, Zhang C, Wei S, Huang Z, Chen J, Wang X (2015) Mutations and amplification of EPSPS gene confer resistance to glyphosate in goosegrass (*Eleusine indica*). *Planta* **242**: 859– 868.

de Carvalho LB, Alves PL, González-Torralva F, Cruz-Hipolito HE, Rojano-Delgado AM, De Prado R, Gil-Humanes J, Barro F, de Castro MD (2012) Pool of resistance mechanisms to glyphosate in *Digitaria insularis*. *Journal of Agricultural and Food Chemistry* **60**: 615– 622.

Han H, Yu Q, Widderick MJ, Powles SB (2016) Target-site EPSPS Pro-106 mutations: sufficient to endow glyphosate resistance in polyploid *Echinochloa colona*? *Pest Management Science* **72**:264–271

Jasieniuk M, Ahmad R, Sherwood AM, Firestone JL, Perez-Jones A, Lanini WT, Mallory-Smith, C, Stednick Z (2008) Glyphosate-resistant Italian ryegrass (*Lolium multiflorum*) in California: distribution, response to glyphosate, and molecular evidence for an altered target enzyme. *Weed Science* **56**: 496–502.

Kaundun SS, Dale RP, Zelaya IA, Dinelli G, Marotti I, McIndoe E, Cairns A (2011) A novel P106L mutation in EPSPS and an unknown mechanism(s) act additively to confer resistance to glyphosate in a South African *Lolium rigidum* population. *Journal of Agricultural and Food Chemistry* **59**: 3227–3233.

Li J, Peng Q, Han H, Nyporko A, Kulynych T, Yu Q, Powles S (2018) Glyphosate resistance in Tridax procumbens via a novel EPSPS Thr-102-Ser substitution. *Journal of Agricultural and Food Chemistry* **66**: 7880–7888.

Nandula VK, Ray JD, Ribeiro DN, Pan Z, Reddy KN (2013) Glyphosate resistance in tall waterhemp (*Amaranthus tuberculatus*) from Mississippi is due to both altered target-site and nontarget-site mechanisms. *Weed Science* **61**: 374–383.

Ng CH, Wickneswari R, Salmijah S, Teng YT, Ismail BS (2003) Gene polymorphisms in glyphosate-resistant and -susceptible biotypes of *Eleusine indica* from Malaysia. *Weed Research* (Oxford) **43**: 108–115.

Perez-Jones A, Park KW, Polge N, Colquhoun J, Mallory-Smith CA (2007) Investigating the mechanisms of glyphosate resistance in *Lolium multiflorum*. *Planta* **226**: 395– 404.

Simarmata M, Penner D (2008) The basis for glyphosate resistance in rigid ryegrass (*Lolium rigidum*) from California. *Weed Science* **56**: 181–188.

Wakelin AM, Preston C (2006) A target-site mutation is present in a glyphosate-resistant *Lolium rigidum* population. *Weed Research* (Oxford) **46**: 432 - 440.

Yu Q, Cairns A, Powles S (2007) Glyphosate, paraquat and ACCase multiple herbicide resistance evolved in a *Lolium rigidum* biotype. *Planta* **225**: 499–513.

Yu Q, Jalaludin A, Han H, Chen M, Sammons RD, Powles SB (2015) Evolution of a double amino acid substitution in the 5-enolpyruvylshikimate-3-phosphate synthase in *Eleusine indica* conferring high-level glyphosate resistance. *Plant Physiology* **167**: 1440–1447.
