# Supplementary figures and images for: Overexpression of improved EPSPS gene results in field level glyphosate tolerance and higher grain yield in rice
Source: Plant Biotechnol J. 2020 Jul 24;18(12):2504–19. doi: 10.1111/pbi.13428 (PMC7680544; doi:10.1111/pbi.13428)

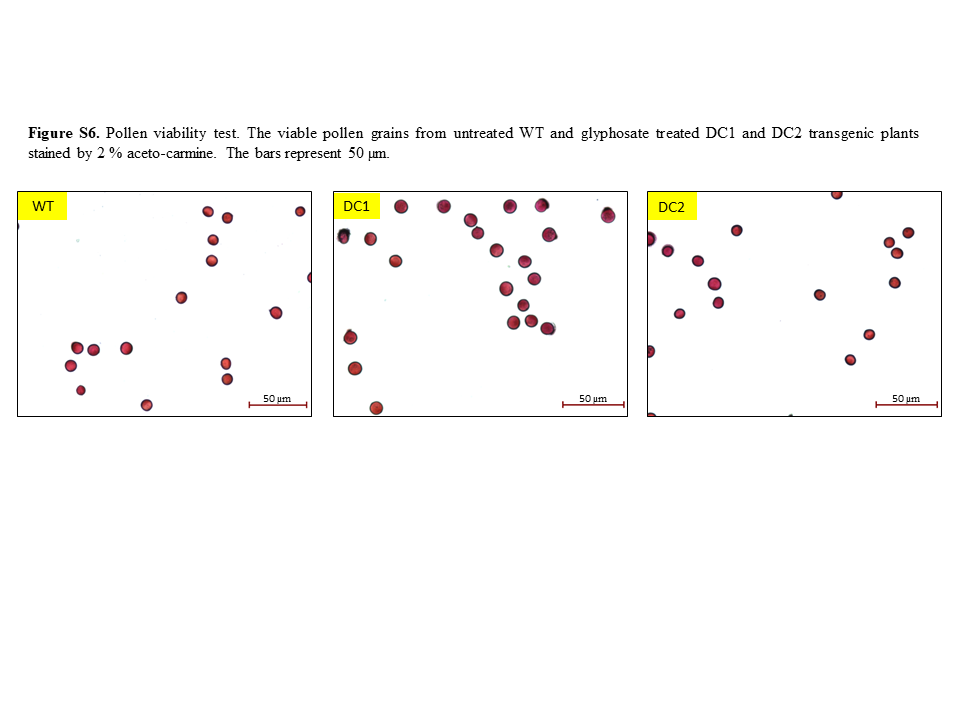

Supplement: Supplementary file 1 — Experimental Procedures. Figure S1. (a) Polynucleotide sequence of rice EPSPS promoter and (b) rice EPSPS terminator. Highlighted nucleotide sequence represents the primer sequence along with restriction sites. Figure S2. (a) Polynucleotide sequence of Zea mays polyubiquitin 1 (ZmUbi) promoter and (b) nopaline synthase gene terminator. Highlighted nucleotide sequence represents the primer sequence along with restriction sites. Figure S3. Polynucleotide DNA sequence of rice EPSP synthase. The blue colour text represent chloroplast transit peptide sequence. Figure S4. Polynucleotide DNA sequence of mutant (T/173/I and P/177/S) TIPS‐OsEPSPS. The blue colour text represents chloroplast transit peptide sequence. The amino acid substitution mutations T/173/I and P/177/S are highlighted in pink and yellow colour respectively. Figure S5. Polynucleotide DNA sequence of mutant (G/172/A, T/173/I and P/177/S) GATIPS‐OsEPSPS. The blue colour text represents chloroplast transit peptide sequence. The amino acid substitution mutations G/172/A, T/173/I and P/177/S are highlighted in red, pink and yellow colour respectively. Figure S6. Pollen viability test. The viable pollen grains from untreated WT and glyphosate treated DC1 and DC2 transgenic plants stained by 2 % aceto‐carmine. The bars represent 50 µm. Figure S7. Relative levels of free aromatic amino acids in rice seeds extracted in Aqueous methanol chloroform (MeOH: CHCl3: H2O (5:2:1)) and analysed by GC‐MS after TBDMS derivatisation. The relative peak abundances of Phenylalanine, Tyrosine and Tryptophan in wild type (WT) and transgenic rice seeds were normalised to L‐norleucine (60 µL of 0.2 mg/mL) as internal standard with abundance set at 100. Table S1. List of primers used in the study Table S2. Glyphosate resistance amino acid substitutions mutations (in EPSPS) identified in resistance‐weed biotypes Table S3. Similarity percentage of protein sequences among various EPSPS enzymes from different organisms. [file PBI-18-2504-s001.zip › pbi13428-sup-0007-FigS6.tif]
